# Study on Dual-Targeted Liposomes Containing Curcumin-Copper Chelate in the Treatment of Triple-Negative Breast Cancer

**DOI:** 10.3390/ph19010135

**Published:** 2026-01-13

**Authors:** Lina Wu, Xueli Guo, Pan Guo

**Affiliations:** 1 State Key Laboratory of Component-Based Chinese Medicine, Tianjin University of Traditional Chinese Medicine, Tianjin 301617, China; wulina13108@163.com (L.W.); 15516164560@163.com (X.G.); 2 State Key Laboratory of Chinese Medicine Modernization, Tianjin University of Traditional Chinese Medicine, Tianjin 301617, China

**Keywords:** breast cancer, curcumin–copper chelate, targeted liposomes, cuproptosis, tumor therapy

## Abstract

**Background**: Triple-negative breast cancer (TNBC) remains primarily treated with chemotherapy due to the lack of effective therapeutic targets, but this approach carries significant systemic toxicity and a high risk of drug resistance. Curcumin (Cur), despite its multifaceted antitumor activity, faces limitations in clinical application due to poor water solubility and weak targeting properties. This study aims to develop a folate/mitochondria dual-targeted curcumin–copper chelate liposome (Cu-Cur DTLPs) formulation that enables copper accumulation within tumor cells and induces copper-mediated cell death, thereby providing an effective and relatively low-toxicity therapeutic strategy for triple-negative breast cancer. **Methods**: Curcumin–copper chelates (Cu-Cur) were first synthesized and characterized using mass spectrometry, NMR, and infrared spectroscopy. Subsequently, dual-targeted liposomes (Cu-Cur DTLPs) were prepared via the thin-film dispersion method, with systematic evaluation of particle size, zeta potential, encapsulation efficiency, and in vitro release profiles. In vitro cytotoxicity was assessed against 4T-1 and MDA-MB-231 cells using the MTT assay. In a 4T-1 tumor-bearing BALB/c mouse model, comprehensive evaluation of targeting efficiency, antitumor efficacy, and mechanisms of action was conducted via in vivo imaging, tumor volume monitoring, immunohistochemistry (detecting FDX1 and DLAT proteins), and TUNEL staining. **Results**: Cu-Cur DTLPs with a uniform particle size of approximately 104.4 nm were successfully synthesized. In vitro and in vivo studies demonstrated that compared to free curcumin and conventional liposomes, Cu-Cur DTLPs significantly enhanced drug accumulation in tumor tissues and exhibited effective tumor growth inhibition. Mechanistic studies confirmed that this formulation specifically accumulates copper ions within tumor cells, upregulates FDX1, promotes DLAT oligomerization, and induces mitochondrial dysfunction, thereby driving copper death. TUNEL staining ruled out apoptosis as the primary mechanism. Safety evaluation revealed no significant toxicity in major organs. **Conclusions**: The Cu-Cur DTLPs developed in this study effectively induce copper-mediated death in TNBC through a dual-targeted delivery system, significantly enhancing antitumor activity with favorable safety profiles. This establishes a highly promising novel nanotherapeutic strategy for TNBC treatment.

## 1. Introduction

### 1.1. Tumors and Its Current Treatment Status

Breast cancer is the most common malignancy in women, and one of the three most common cancers worldwide, seriously threatening women’s health and quality of life [1]. Triple-negative breast cancer (TNBC) is characterized by the lack of expression of estrogen receptor, progesterone receptor, and human epidermal growth factor receptor 2. It is the most aggressive molecular subtype among breast tumors [2]. Because targeted therapy for TNBC is challenging, Chemotherapy, radiation therapy, and surgery are the common therapies for TNBC. However, these treatment methods often come with severe toxic side effects and easily develop drug resistance, leading to poor prognosis for patients [3]. In recent years, although novel therapies such as immunotherapy and PARP inhibitors have made some progress in TNBC treatment, the overall efficacy still needs improvement [4]. Therefore, developing new, highly effective, and low-toxicity treatment strategies for TNBC is urgently needed and is of great significance for improving the survival rate and quality of life for TNBC patients.

### 1.2. Overview of Cuproptosis Mechanism

Cuproptosis is a newly identified form of cell death, this study opens new avenues for developing tumor therapy [5]. Mechanistically, cuproptosis results from Cu-induced aggregation of dihydrolipoamide S-acetyltransferase, correlated with the mitochondrial tricarboxylic acid cycle and the loss of iron–sulfur cluster proteins, ultimately resulting in proteotoxic stress and triggering cell death. Research indicates that copper ions play a crucial role in the proliferation, invasion, and metastasis of tumor cells. Tumor cells often exhibit abnormal copper metabolism characteristics, showing more active uptake and utilization of copper ions compared to normal cells [6]. This difference provides a theoretical basis for developing tumor treatment strategies based on cuproptosis mechanisms. It is expected that by precisely regulating copper ion levels within tumor cells, specific killing of tumor cells can be achieved while reducing damage to normal cells.

### 1.3. Association Between Breast Cancer and Copper Metabolism

An increasing body of literature supports the role of copper homeostasis imbalance in tumor proliferation and metastasis. Studies have shown that breast cancer cells typically exhibit abnormal copper metabolism characteristics, such as increased copper uptake, elevated copper content, and abnormal expression of copper metabolism-related genes [7]. These abnormal copper metabolic activities are closely related to the proliferation, angiogenesis, metastasis, and drug resistance of breast cancer cells [8]. Therefore, regulating the copper metabolism of breast cancer cells, particularly by increasing intracellular copper ion concentration to induce cuproptosis, may become an effective anti-tumor strategy [9].

### 1.4. Synergistic Effects of Curcumin and Copper

Modern research has found that curcumin possesses multiple biological activities, such as antioxidant, anti-inflammatory, antibacterial, and potential anti-tumor effects [10]. Notably, in the presence of copper ions, curcumin exhibits a unique dual antioxidant/pro-oxidant sword-like effect. On one hand, under physiological conditions, curcumin acts as an antioxidant, scavenging free radicals in the body and protecting cells from oxidative damage. On the other hand, under specific conditions such as the tumor microenvironment, curcumin can serve as a copper ion carrier [11,12], promoting intracellular copper accumulation and enhancing the toxic effects of copper ions on tumor cells [13,14]. Studies indicate that the anti-tumor activity of curcumin is partly attributed to its copper-chelating ability, as the formed copper-curcumin complex can selectively target cancer cells, induce apoptosis, and inhibit tumor growth [13,15]. This synergistic effect with copper ions gives curcumin great potential in tumor therapy, providing new insights for developing novel anti-tumor drugs.

### 1.5. Delivery Effect of Dual-Targeted Liposomes

Liposomes, as a nanomedicine carrier, possess excellent biocompatibility, drug encapsulation capacity, and modifiability, making them highly attractive in tumor-targeted therapy [16]. Compared to traditional liposomes, targeted liposomes can specifically recognize receptors overexpressed on tumor cell surfaces, guiding precise binding to tumor cells, increasing drug accumulation in tumor tissues, and thereby improving therapeutic efficacy while reducing toxic side effects on normal tissues [17]. They can also enhance cellular uptake; through receptor-mediated endocytosis, targeted liposomes can more efficiently enter tumor cells, delivering drugs to intracellular target sites such as cell nuclei and mitochondria, thus enhancing the killing effect of the drug [18]. This study selected two targeting ligands: DSPE-mPEG2k-FA (folate ligand) and DSPE-mPEG2k-MLS (subcellular-mitochondria targeting peptide), aiming to achieve dual targeting of tumor cells and their intracellular mitochondria, and to combine with the cuproptosis mechanism to exert a stronger anti-tumor effect. Folic acid (FA) is a water-soluble vitamin that plays a crucial role in cell growth and proliferation. Many tumor cells, including breast cancer cells, have a significantly higher demand for folic acid due to their rapid proliferation, leading to overexpression of folate receptors (Folate Receptor, FR) [19,20]. By coupling folic acid with DSPE-mPEG2k to form DSPE-mPEG2k-FA, liposomes can selectively bind to folate receptors on the surface of tumor cells, enter cells through receptor-mediated endocytosis, thereby enhancing drug accumulation in tumor sites [21]. Mitochondria are the energy factories of cells and key regulatory centers for apoptosis, and they are also the primary sites where cuproptosis occurs [22]. Triple-negative breast cancer cells typically exhibit stronger invasiveness and metastatic capabilities, with more pronounced mitochondrial dysfunction, making them more sensitive to drugs with mitochondrial targeting functions [23]. DSPE-mPEG2k-MLS is a DSPE-mPEG2k derivative conjugated with a mitochondria-localizing sequence (MLS), which uses MLS as a mitochondria-targeting ligand to actively guide liposomes into cells and further target mitochondria, delivering drugs to the vicinity of mitochondria [24,25]. By covalently conjugating DSPE-mPEG2k-FA and DSPE-mPEG2k-MLS to the liposome surface, dual-targeting liposomes are constructed, which can achieve synergistic targeting of tumor cells and their intracellular mitochondria. This dual-targeting strategy is expected to overcome the limitations of conventional treatments, enhance drug efficacy while reducing toxic side effects on normal tissues, and provide a new approach for the treatment of triple-negative breast cancer.

### 1.6. Design Concept

Based on the above research background, this study proposes a novel breast cancer targeted therapy strategy: curcumin is chelated with copper ions to form curcumin–copper chelate/Cu-Cur, which is designed and constructed as curcumin–copper chelate double-targeting liposomes Cu-Cur DTLPs. First, folate ligands guide liposomes to bind to the surface of tumor cells and enter the cells through endocytosis. Then, mitochondria-targeting peptides guide liposomes to further target mitochondria, release curcumin–copper chelate in the energy metabolism center of tumor cells, and increase the concentration of curcumin and copper ions in mitochondria, thereby more effectively inhibiting mitochondrial metabolism, selectively inducing copper death in tumor cells, and maximizing the anti-tumor effect of drugs, so as to achieve efficient and low-toxicity anti-tumor treatment effects. By systematically studying the synthesis, characterization, in vitro cell experiments, in vivo targeting, and in vivo anti-tumor effects, we will comprehensively evaluate their feasibility and effectiveness as a new treatment strategy for breast cancer, and provide new theoretical basis and potential schemes for the clinical treatment of triple-negative breast cancer.

## 2. Results

### 2.1. Synthesis and Characterization of Curcumin–Copper Chelate (Cu-Cur)

#### 2.1.1. Synthesis Results

By collecting the final samples and weighing them, the filtrate product yielded 50–60% based on the initial total mass of reagents. It appeared as an orange-yellow crystalline powder. The filter residue product formed a reddish-brown powder.

#### 2.1.2. Analysis of EPR Results for Filtrate and Filter Residue

**Figure 1:**
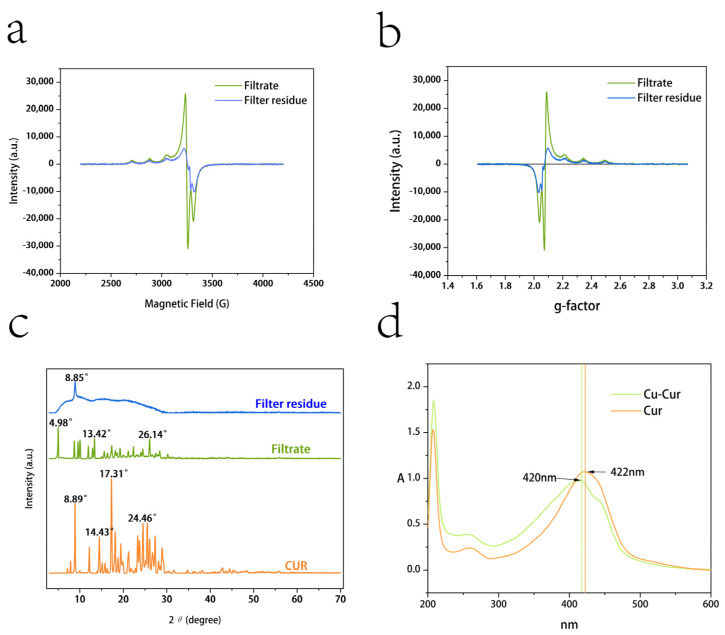
(**a**) EPR results for filtrate and filter residue products: magnetic field strength versus signal absorption intensity; (**b**) EPR results for filtrate and filter residue products: g-value versus signal absorption intensity. (**c**) XRD patterns of filtrate products, filter residue products, and curcumin. (**d**) UV spectra of Cu-Cur and Cur.

Figure 1a shows the variation in absorption signal intensity of the filtrate and filter residue products with changes in applied magnetic field strength. When the applied magnetic field strength satisfies resonance conditions, electrons absorb microwave energy at a fixed frequency, resulting in signal peaks on the spectrum. The main peak of the filtrate product corresponds to a magnetic field strength of approximately 3234 G, while the secondary peak corresponds to approximately 3056 G. The narrow line width indicates a relatively homogeneous molecular environment, consistent with the characteristics of a mononuclear copper complex. The main peak of the filter residue product corresponds to a magnetic field strength of approximately 3220 G, while the secondary peak corresponds to approximately 3048 G. The signal peak width is significantly broadened, and the peak shape is irregular, reflecting the presence of complex electron exchange pathways within the layered polymer.

By calculating the Landé g-factor (g value) and hyperfine coupling constant (*A* value), we determine the relevant parameters for EPR experiments involving filtrate and filter residue products, analyzing the differences between the two.

The g-value is calculated using the following formula:

(1) $$g=\frac{hv}{\beta B}$$

The *A*-value is calculated using the following formula:

(2) $$A=\frac{g\beta \Delta B}{hv}$$

where *h* = 6.626 × 10^−34^ J·s; *ν* = 9.5 GHz = 9.5 × 10^9^ Hz; *β* = 9.274 × 10^−24^ J/T; *B* [mT] denotes magnetic field strength, measured in millitesla.

The comprehensive EPR parameters for filtrate products and filter cake products are shown in Table 1.

Figure 1b shows the variation in g-values with absorption signal intensity. The main peaks of the filtrate product and the filter residue product are close to g = 2.10 and g = 2.03, respectively. The g// value of the filtrate product indicates that copper ions are in a strong coordination field, potentially forming a mononuclear complex. The peak is narrow with high signal intensity (approximately 30,000 a.u.), reflecting uniform molecular arrangement and crystallinity, consistent with the orange-yellow crystalline appearance of the filtrate product. The g// < g**⊥** value for the filtrate product aligns with the characteristics of a planar quadrilateral structure. In such structures, the copper ion typically resides within the plane formed by four ligands, featuring a symmetrical coordination environment with uniform electron distribution [26]. A**⊥** = 54 mT represents a large hyperfine coupling constant, indicating strong covalent interactions between copper ions and ligands in the equatorial plane, consistent with typical copper ion-organic ligand complexes. A// = 51 mT indicates weak coordination along the axial direction. The structure of the filtrate product likely involves bidentate chelation between the copper ion and the β-diketone group of curcumin [27,28]. The lower g-value of the filter residue product suggests the presence of metallic copper aggregates in the powder. Their absorption peaks exhibit broad signals with low intensity (approximately 10,000 a.u.), reflecting intermolecular super-exchange interactions and disordered stacking. The filter residue may also contain polycoordinate coordination and layered polymeric structures; however, due to solubility limitations, its value for pharmacodynamic studies is low [29].

#### 2.1.3. XRD Results

As shown in Figure 1c, the XRD pattern of curcumin exhibits a series of sharp and intense diffraction peaks, indicating that curcumin is a crystalline substance. Characteristic peaks are concentrated in the low-angle range (e.g., 10–30°), corresponding to curcumin’s specific crystal structure. Compared to the filtrate product, curcumin exhibits denser and more intense diffraction peaks, indicating more regular molecular arrangement. The positions of high-intensity peaks in the data (e.g., 17.31°, 24.46°) can serve as fingerprint characteristics for curcumin crystals [30,31].

The XRD pattern of the filtrate product exhibits multiple sharp diffraction peaks, particularly in the low-angle region (2θ < 20°), indicating the presence of crystalline phases. In the high-angle region (2θ > 20°), the intensity of diffraction peaks gradually diminishes, suggesting reduced crystallinity. Compared to curcumin’s diffraction peaks, some peaks exhibit positional shifts. These peaks suggest the potential presence of a copper ion complex formed through the reaction between copper ions and curcumin. The peak intensity and position indicate the filtrate contains a high proportion of crystalline material, whose primary component is likely a dissolved copper ion complex possessing a crystalline structure. Its high symmetry and regularity align with the symmetric electronic environment observed in EPR data. The excellent crystallinity supports its high solubility and antioxidant activity [32,33].

The XRD pattern of the filter residue exhibits broad diffraction peaks with low intensity, indicating poor crystallinity. Strong, broad, doughnut-shaped peaks appear near the low-angle region (2θ < 10° and 20°), likely associated with an amorphous or microcrystalline structure [34]. The filter residue product likely represents a layered polymer, where Cu-O-Cu bridging structures induce disordered intermolecular stacking. Its amorphous or microcrystalline nature aligns with the anisotropic electronic environment observed in EPR data. The low crystallinity and disordered stacking of amorphous or microcrystalline layered polymers result in poor solubility, potentially conferring sustained-release material properties [35,36].

Following the reaction between curcumin and copper acetate, a portion formed soluble copper complexes (evidenced by sharp diffraction peaks in the filtrate product), while another portion yielded low-crystalline or amorphous solid residues (evidenced by broad peaks in the filter residue product). This result aligns with the EPR findings, indicating that the filtrate product should be collected for subsequent experimental procedures.

#### 2.1.4. Ultraviolet Spectra of Cu-Cur

As shown in Figure 1d, curcumin exhibits a maximum UV absorption wavelength at 422 nm, while Cu-Cur shows a maximum absorption at 420 nm. The chelate exhibits a slight blue shift compared to the former. Additionally, a distinct shoulder peak is observable in the spectrum for Cu-Cur, which provides evidence for the chelation reaction between curcumin and copper ions [37].

#### 2.1.5. Infrared Spectroscopy Results for Cu-Cur

**Figure 2:**
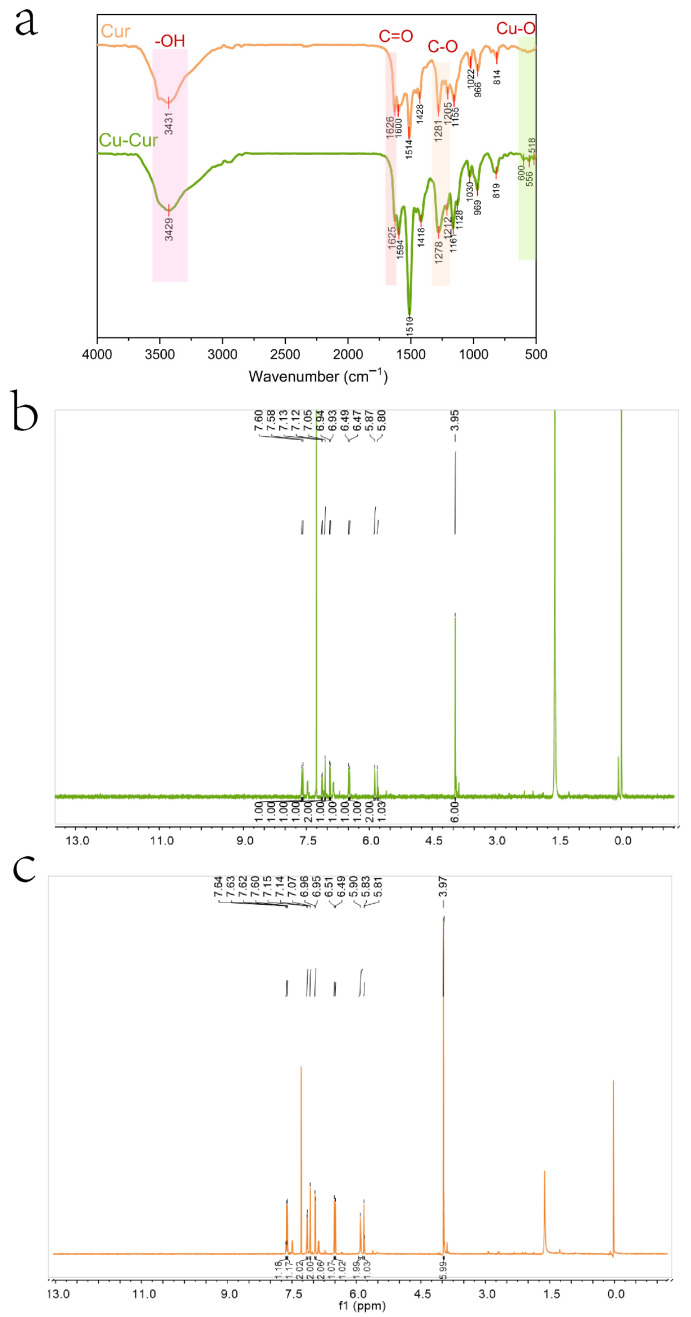
(**a**) Infrared Spectra of Cur and Cu-Cur. (**b**) Nuclear Magnetic Resonance Hydrogen Spectrum of Cu-Cur (**c**) Nuclear Magnetic Resonance Hydrogen Spectrum of Cur.

As shown in Figure 2a, the infrared spectrum of curcumin exhibits a prominent broad peak at 3200–3600 cm^−1^, characteristic of the hydroxyl ν(-OH) stretching vibration, indicating the presence of a phenolic hydroxyl group in the curcumin molecule. Weak absorption peaks are observed in the 3100–3000 cm^−1^ range, potentially corresponding to the ν(=C-H) stretching vibration of hydrogen atoms on the alkene bond, indicating the presence of aromatic rings or alkenes in the molecule. A strong absorption peak near 1626 cm^−1^ is assigned to the ν(C=O) stretching vibration of the β-diketone conjugated system, one of the key characteristics of curcumin molecules [38]. Peaks at 1600 cm^−1^ and 1514 cm^−1^ correspond to the ν(C=C) stretching vibration of the aromatic ring, indicating the presence of a benzene ring structure in curcumin. A weak peak at 1428 cm^−1^ may be associated with ν(C-H) bending vibrations. Absorption peaks around 1200 cm^−1^ (1281 cm^−1^ and 1155 cm^−1^) are typically related to ν(C-O) stretching vibrations of phenolic hydroxyl groups or ether bonds, consistent with curcumin’s molecular structure. The region between 500 and 1000 cm^−1^ contains characteristic vibrations of curcumin, possibly related to ν(C-H) bending and ν(C-O-C) stretching vibrations, further confirming the presence of aromatic rings [38].

The infrared spectrum of the chelate also exhibits a broad peak at 3200–3500 cm^−1^, indicating that the hydroxyl group remains present after chelation. The absorption peak at 1625 cm^−1^ is attributed to the stretching vibration of the carbonyl ν(C=O). Compared to the carbonyl shift in curcumin, it exhibits a slight red shift and significantly reduced intensity, indicating that the β-diketone has converted to an enol form. This conversion enhances electron delocalization, This may result from coordination of both ketone groups in curcumin with copper ions, causing a shift in the vibration frequency of the carbonyl group [38]. Multiple moderate-intensity absorption peaks appearing in the 1500–1600 cm^−1^ range indicate that the aromatic ring structure in curcumin remains intact after the reaction, though its stretching vibration peak exhibits slight changes, possibly due to alterations in electron density. A weak peak near 1400 cm^−1^ likely relates to alterations in the aromatic ring’s electronic environment following metal coordination. Changes in the stretching vibrations of the ν(C-O) bond at 1278 cm^−1^ and 1212 cm^−1^ further support oxygen atom participation in coordination. Weak absorption peaks observed in the low-wavenumber region (400–600 cm^−1^) may represent characteristic vibrations of metal-oxygen coordination bonds (e.g., Cu-O), providing further evidence that copper ions form coordination bonds with oxygen atoms in curcumin [39].

Based on spectral characteristics and the chemical properties of curcumin, it is speculated that the carbonyl group within the β-diketone structure of the curcumin molecule may serve as a coordination site to form a tetracoordinate organometallic complex with copper ions. Given that the molar ratio of curcumin to copper acetate during synthesis was 2:1, a bidentate coordination structure may form, wherein a single copper ion center coordinates with two curcumin molecules simultaneously. This is consistent with previous EPR and XRD results.

#### 2.1.6. NMR Spectral Analysis Results of Cu-Cur

Figure 2b,c display the nuclear magnetic resonance hydrogen spectra of Cu-Cur and curcumin, respectively, with their respective analyses. The 1H NMR spectrum of Cu-Cur is analyzed as follows: 1H NMR (600 MHz, CDCl_3_), δ 7.60 (s, 1H), 7.58 (s, 1H), 7.13 (s, 1H), 7.12 (s, 1H), 7.05 (s, 2H), 6.94 (s, 1H), 6.93 (s, 1H), 6.49 (s, 1H), 6.47 (s, 1H), 5.87 (s, 2H), 5.80 (s, 1H), 3.95 (s, 6H).

The 1H NMR spectrum of Cur is analyzed as follows: 1H NMR (600 MHz, CDCl_3_): δ 7.62 (dd, J = 15.8, 9.2 Hz, 2H), 7.15 (d, J = 8.1 Hz, 2H), 7.07 (s, 2H), 6.96 (d, J = 8.1 Hz, 2H), 6.50 (d, J = 15.7 Hz, 2H), 5.90 (s, 2H), 5.82 (d, J = 8.4 Hz, 1H), 3.97 (s, 6H).

Comparing the data from both studies reveals that after curcumin chelates copper ions, the displacement of each hydrogen atom decreases to varying degrees, shifting toward the high-field region. Curcumin contains coordination sites with oxygen atoms capable of providing lone-pair electrons. When chelating with copper ions, the copper ions accept the electron pairs supplied by the curcumin ligands, altering the electron density distribution around certain atoms within curcumin [38]. Since copper ions exhibit lower electronegativity than atoms like oxygen, electrons shift toward certain functional groups within the curcumin molecule. This enhances the electron density around hydrogen nuclei bonded to these groups, intensifying the shielding effect. Consequently, chemical shifts migrate toward higher field values, resulting in reduced shift data [40]. Regarding coupling changes, the hydrogen nuclei that were originally coupled undergo alterations after copper ion chelation. The coupling constants decrease or even disappear, with most multi-peaks transforming into single peaks. Additionally, the peak shapes reveal that the Cu-Cur peak is broader and exhibits more diffuse signals. These changes may be related to the formation of metal coordination bonds. Following metal coordination, the dynamic exchange effects or hydrogen nucleus coupling between the originally coupled hydrogen nuclei are disrupted, leading to a transition from multiple peaks to a single peak [41]. The formation of metal coordination bonds may restrict the molecular degrees of freedom, reducing the elasticity of overall molecular motion and affecting hydrogen nucleus relaxation processes. Typically, this results in prolonged spin-lattice relaxation times and shortened spin-spin relaxation times. The latter may explain the increased peak width observed in the Cu-Cur hydrogen spectrum compared to curcumin [42].

#### 2.1.7. Mass Spectrometry Results

**Figure 3:**
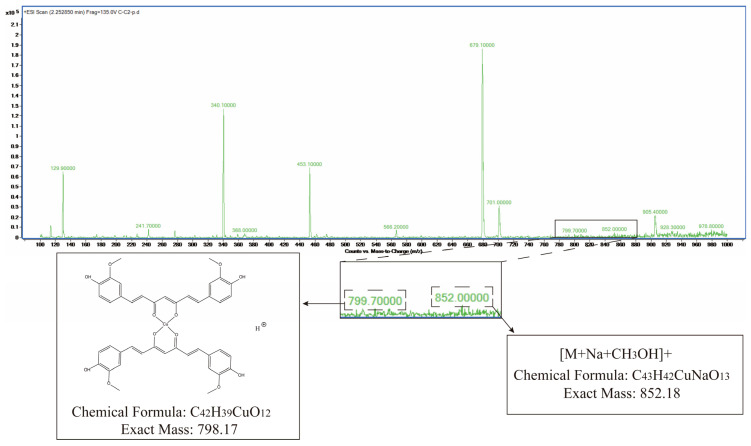
Mass spectrometry results for Cu-Cur.

Mass spectrometry analysis of the absorption peak at 2.258 min revealed MS(ESI): *m*/*z* = 799.7[M + H]+ and MS(ESI): *m*/*z* = 852.0[M + Na + CH3OH]+, consistent with the theoretical molecular mass of Cu-Cur, confirming the successful synthesis of Cu-Cur (Figure 3).

#### 2.1.8. Melting Point Determination Results

Table 2 presents the melting point differences between filtrate products and filter residue products. It can be observed that the filtrate products exhibit both initial melting and final melting phenomena, with relatively concentrated melting point ranges. In contrast, the filter residue products did not show distinct initial or final melting changes during measurement; the three measured temperatures were relatively close and significantly higher than the melting point range of the filtrate products. This result indicates that the filtrate products and filter residue products possess markedly different material properties.

The melting points of filtrate products and filter residue products exhibit differences, primarily related to their material composition and structure. Differences in Composition: The filtrate product likely consists primarily of copper chelates formed during the reaction. These chelates possess relatively regular and uniform structures, endowing them with well-defined melting points. When heated within a specific temperature range, the intermolecular forces between the chelate molecules break down, resulting in a melting process characterized by an initial melting temperature followed by a final melting temperature. In contrast, the residue product has a complex composition, potentially containing unreacted curcumin, its aggregates, and trace amounts of copper ion complexes. The presence of unreacted curcumin in the residue contributes to its impurity, with different components exhibiting distinct melting points, making it difficult to observe a unified initial and final melting phenomenon. Different Crystal Structures: The filtrate product exhibits multiple sharp diffraction peaks in its XRD pattern, particularly in the low-angle region (2θ < 20°), indicating the presence of crystalline phases with good crystallinity. The ordered crystal structure enables regular molecular arrangement, leading to a sequential phase transition upon heating and a distinct melting range. In contrast, the XRD pattern of the residue product exhibits broad, low-intensity peaks, indicating poor crystallinity. A strong broad peak appears in the low-angle region (2θ < 10°), characteristic of amorphous or microcrystalline structures. This amorphous or microcrystalline structure results in inconsistent intermolecular forces, preventing simultaneous phase transitions during heating. Consequently, distinct initial and final melting points are not observed; instead, a nearly uniform melting characteristic emerges at higher temperatures.

#### 2.1.9. Thermogravimetric Analysis (TGA) Results

The TGA curves of curcumin and its copper chelates primarily differ in their thermal stability and decomposition patterns.

**Figure 4:**
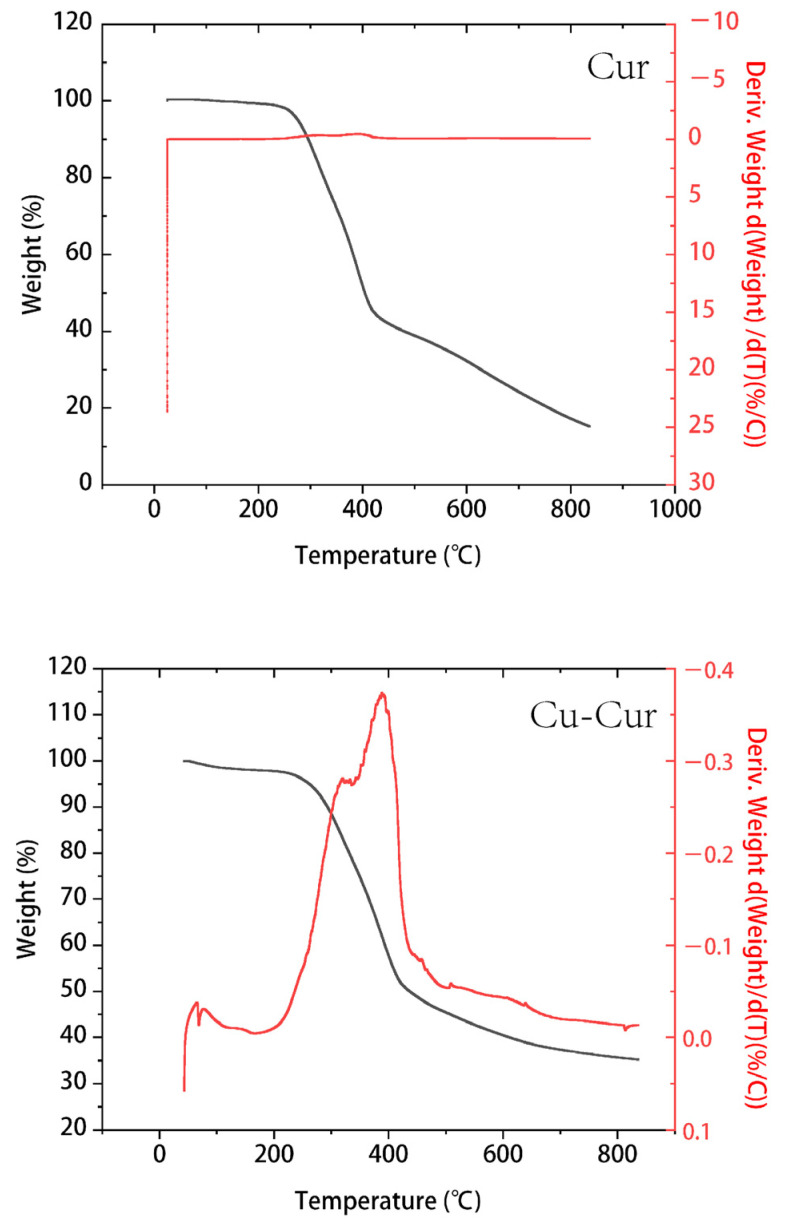
Thermogravimetric curve of Cur and Cu-Cur.

Curcumin (Figure 4): TGA typically reveals a multi-step decomposition process. The blue curve represents the mass change in curcumin with temperature. At lower temperatures (approximately 0–200 °C), the mass remains stable around 100%, indicating high stability of curcumin within this temperature range. Starting around 200 °C, mass decreases rapidly. By approximately 600 °C, the rate of mass loss levels off, with only a small residual mass remaining. This indicates decomposition occurs within this temperature range. The green curve represents the derivative of the blue curve, reflecting the rate of mass change. During the rapid mass loss phase, its absolute values are significantly higher.

Cu-Cur (Figure 4): Compared to pure curcumin, the copper chelate exhibits superior thermal stability. The primary decomposition step shifts to a higher temperature range, with a relatively gradual mass loss trend beginning around 200 °C. A distinct acceleration phase in mass loss occurs between 300 and 400 °C, followed by a gradual decrease in the rate of mass loss. Ultimately, the residual mass exceeds that of curcumin, indicating that copper coordination enhances curcumin’s molecular structure, making it more resistant to thermal degradation. The DTG curve of the chelate also exhibits more distinct peaks, indicating that Cu-Cur possesses a distinct decomposition phase compared to the broader degradation observed in pure curcumin.

#### 2.1.10. Logarithm of Octanol–Water Partition Coefficient (logP) Determination Results for Cu-Cur

**Figure A1:**
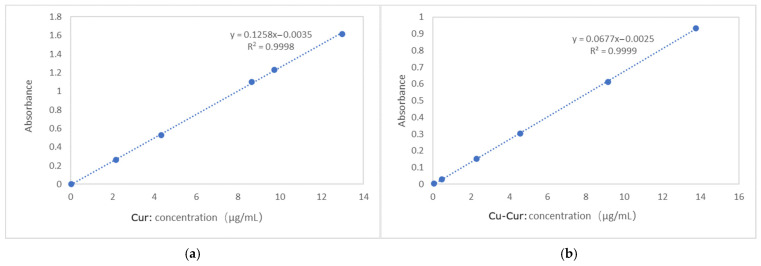
Standard Curve for Cur (**a**) and Cu-Cur (n-Octanol Solubility) (**b**).

Standard curve for curcumin (n-octanol): y = 0.1258x − 0.0035, R^2^ = 0.9998, Standard curve for Cu-Cur (n-octanol): y = 0.0677x − 0.0025, R^2^ = 0.9999 (Figure A1). The volume ratios of n-octanol-saturated water to water-saturated n-octanol from three parallel experiments were recorded as 1:2, 1:1, and 2:1, denoted, respectively, as 1-1~3, 2-1~3, and 3-1~3.

As shown in Table A1 and Table A2, the logP value of curcumin is 3.38, which is consistent with the range reported in the literature [37]. The logP value of Cu-Cur is 2.08, lower than that of free curcumin. The decrease in logP indicates that the hydrophilicity of the curcumin molecule increases after chelation with copper ions. This may result from the coordination of copper ions with the hydroxyl and ketone groups in the curcumin molecule, enhancing molecular polarity and interactions with water molecules. This change in hydrophilicity may influence the distribution, absorption, and metabolic behavior of Cu-Cur within the body, potentially increasing its solubility and bioavailability in aqueous environments.

### 2.2. Evaluation of Cu-Cur Dual-Targeted Liposomes

#### 2.2.1. Preparation of Liposomes and Investigation of Particle Size, PDI, and Zeta Potential

**Figure 5:**
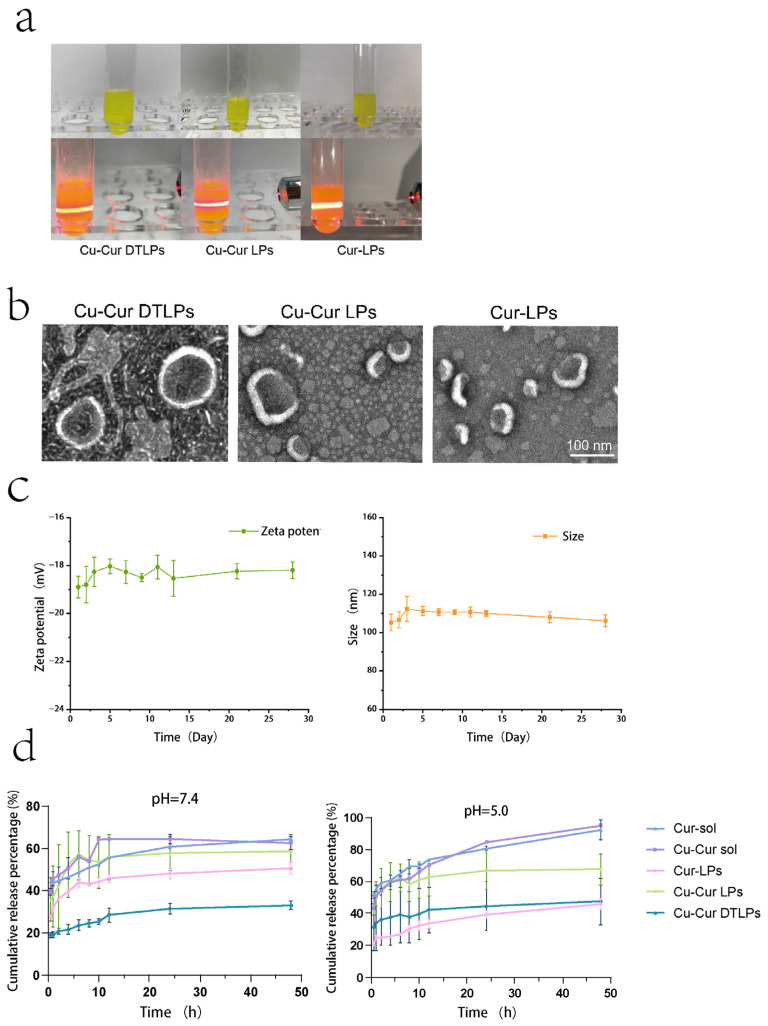
(**a**) Appearance and Tyndall Effect of Cu-Cur DTLPs, Cu-Cur LPs, and Cur-LPs after 10-fold Dilution. (**b**) TEM images of Cu-Cur DTLPs, CuCur LPs, and Cur-LPs. (**c**) Stability of Cu-Cur DTLPs over 28 days at 4 °C. Data are shown as mean ± SD (*n* = 3). (**d**) In vitro release profile (37 °C). Data are shown as mean ± SD (*n* = 3).

The prepared Cu-Cur DTLPs appear as an orange-yellow suspension, Cu-Cur LPs as a bright yellow suspension, and Cur-LPs as a yellow suspension. When illuminated with a red laser pointer, all prepared liposomes exhibit a distinct red pathway and demonstrate the Tyndall effect, as shown in Figure 5a.

**Figure A2:**
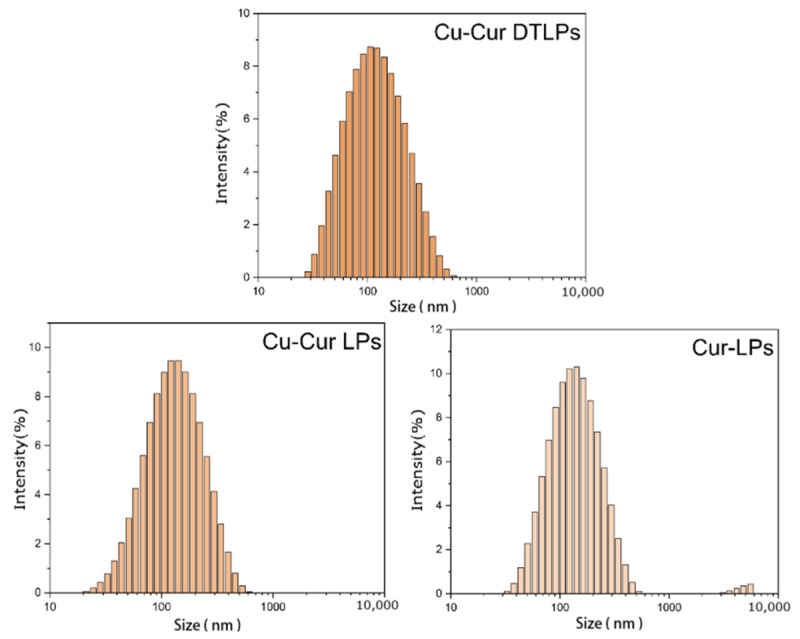
Particle size distribution of Cu-Cur DTLPs, Cu-Cur LPs, and Cur-LPs.

The particle size, PDI, and Zeta potential of Cu-Cur DTLPs, Cu-Cur LPs, and Cur-LPs were determined, with results shown in Table 3 and Figure A2. The results indicate that the particle size of Cu-Cur DTLPs is approximately 104.4 nm, while that of Cu-Cur LPs and Cur-LPs is approximately 117.5 nm and 126.3 nm, respectively. The potential of Cu-Cur DTLPs was −19.1 mV, while Cu-Cur LPs and Cur-LPs exhibited potentials of −17.4 mV and −17.8 mV, respectively.

#### 2.2.2. TEM Results of Drug-Loaded Liposomes

As shown in Figure 5b, transmission electron microscopy images reveal that the prepared Cu-Cur DTLPs exhibit uniform spherical morphology with distinct lipid bilayers. Compared to Cu-Cur LPs, the introduction of the targeting ligand enhances the morphological stability of the liposomes, likely due to a more uniform charge distribution on their surfaces. When comparing Cu-Cur LPs to Cur-LPs, the latter form liposomes with greater size uniformity. None of the three lipid particle groups exhibited noticeable agglomeration, consistent with the well-dispersed appearance observed in actual preparations.

#### 2.2.3. Stability Study of Drug-Loaded Liposomes

Table A3 and Table A4 and Figure 5c illustrate the particle size and zeta potential changes in the targeted drug-loaded liposomes over one month. Calculated RSD values <3% indicate that the prepared Cu-Cur DTLPs exhibit excellent stability, with no significant accumulation or aggregation of liposomes in this system. The stable negative surface charge (Zeta potential < −30 mV) enables avoidance of rapid clearance by the reticuloendothelial system (RES) through electrostatic repulsion, prolonging the in vivo circulation half-life and thereby enhancing EPR effect accumulation at tumor sites. The long-term stability of Cu-Cur DTLPs in terms of particle size, potential, and dispersibility validates their feasibility as an efficient drug delivery system from a physicochemical perspective.

#### 2.2.4. Determination of Drug Loading Capacity and Encapsulation Efficiency of Drug-Loaded Liposomes

The results for the drug loading capacity and encapsulation efficiency of the three sets of liposomes are shown in Table 4. The results indicate that chelating Cur with copper ions significantly enhances the encapsulation efficiency, while the targeting ligand also improves both encapsulation efficiency and drug loading capacity to a certain extent.

#### 2.2.5. In Vitro Release Study of Drug-Loaded Liposomes over 7 Days

A pH 7.4 buffer solution simulates physiological conditions, while a pH 5.0 buffer solution mimics the tumor microenvironment. Under both conditions, the release rates of Cu-Cur and Cur prepared as liposomes were significantly lower than those of the pure drug solutions. The release rates of Cu-Cur and Cur suspensions were higher under acidic conditions, approaching 100%, whereas the cumulative release percentage under neutral conditions could only reach 60%. For Cur-LPs and Cu-Cur LPs, the latter exhibited higher release rates across different media. This may result from increased hydrophilicity after Cur chelation with copper ions, facilitating easier release into buffers. After incorporating folate ligands and mitochondrial ligands into the membrane material, the prepared Cu-Cur DTLPs exhibited lower release rates compared to Cu-Cur LPs without ligands. Furthermore, Cu-Cur DTLPs demonstrated relatively higher release rates in pH 5.0 media. The release profiles of the prepared liposomes under different formulations in various media all exhibited slow-release characteristics, with gradual increases and no significant slope.

### 2.3. In Vitro Cellular Studies of Targeted Drug-Loaded Liposomes

#### Cytotoxicity Evaluation Results of Different Drug-Loaded Liposomes

**Figure 6:**
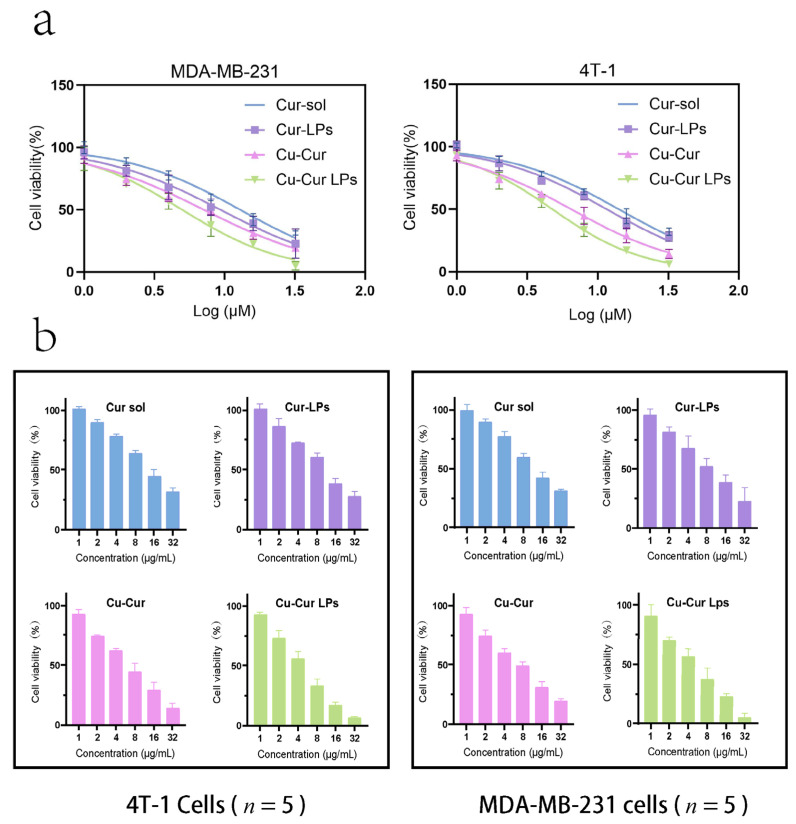
(**a**) Cell survival rate fitting curves of 4T-1 cells and MDA-MB-231 cells under the effects of different group formulations. Data are shown as mean ± SD (*n* = 5). (**b**) In Vitro Cytotoxicity of Different Concentrations of the Formulation on 4T-1 Cells and MDA-MB-231 cells. Data are shown as mean ± SD (*n* = 5).

As shown in Figure 6a, cell viability gradually decreased across all groups with increasing drug concentrations, exhibiting a dose-dependent relationship. For 4T-1 cells, the IC_50_ values for Cur sol, Cur-LPs, Cu-Cur sol, and Cu-Cur LPs were 14.09, 11.57, 6.48, and 4.77, respectively. For MDA-MB-231 cells, the IC_50_ values for Cur sol, Cur-LPs, Cu-Cur sol, and Cu-Cur LPs were 12.90, 9.34, 7.11, and 4.99, respectively. The Cu-Cur LPs group exhibited the lowest IC_50_ value, indicating the strongest antitumor effect, followed by the Cu-Cur sol group. The Cur-LPs and Cur sol groups demonstrated the weakest toxicity.

Figure 6b demonstrate the cytotoxic effects of four formulations on 4T-1 and MDA-MB-231 cells at different concentrations. Cell viability decreased progressively with increasing concentrations across all groups. The Cu-Cur LPs group exhibited significant inhibitory effects across all concentrations, with higher concentrations demonstrating more pronounced suppression. The Cu-Cur group showed weaker inhibition, while the Cur solution group and Cur-LPs group exhibited no significant difference in inhibitory effects. This indicates that the formation of copper chelates significantly enhances curcumin’s antitumor activity, and the liposome carrier system further improves drug delivery efficiency. The Cu-Cur LPs group exhibited similar effects across different breast cancer cell lines, validating its broad-spectrum antitumor activity with a dose-dependent response.

### 2.4. In Vivo Targeting Studies of Drug-Loaded Liposomes

#### 2.4.1. Results of Dir Content Determination in Each Group

Based on the measured fluorescence intensities of each group, the corresponding Dir content was calculated, as shown in Table 5, including both the preparation concentration and the actual administered concentration.

#### 2.4.2. In Vivo Targeting and Tissue Distribution Results

**Figure 7:**
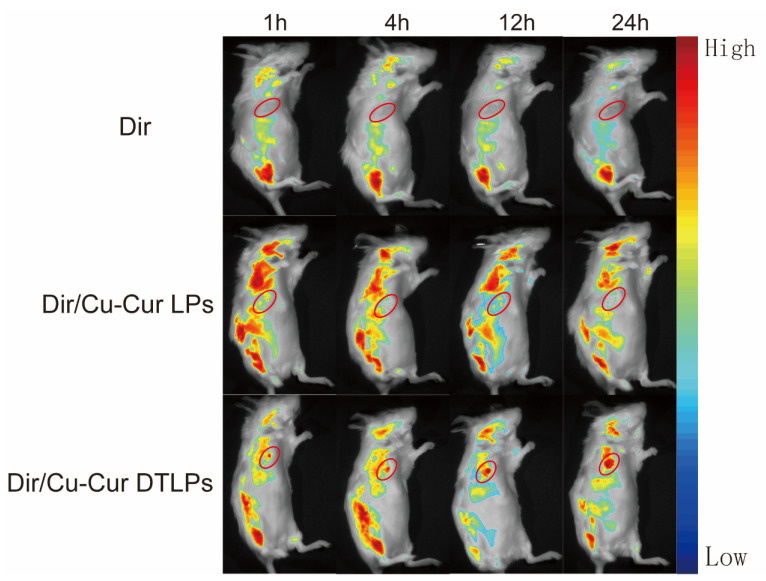
In vivo imaging results within 24 h for each formulation group. The red circle indicates the location of the tumor.

Imaging results at different time points following tail vein administration of each formulation group are shown in Figure 7.

In the tumor fluorescence distribution experiment, all three groups of mice exhibited detectable fluorescence signals at the tumor sites within one hour, indicating that all three formulations began distributing to the tumor sites shortly after administration. Among them, the Dir/Cu-Cur DTLPs group and the Dir/Cu-Cur DTLPs group showed relatively stronger fluorescence signals at the tumor sites, suggesting that these two lipid-based formulations may possess a certain degree of tumor enrichment capability even in the early stages. After 4 h, the tumor fluorescence signal in the Dir solution group diminished, likely due to faster metabolism of the Dir solution in vivo or relatively rapid clearance from the tumor site. In contrast, the tumor fluorescence signals in the Dir/Cu-Cur DTLPs group and the Dir/Cu-Cur DTLPs group both increased, indicating continued accumulation of the lipid formulations at the tumor site over time. The Dir/Cu-Cur DTLPs group exhibited relatively higher fluorescence intensity at this time point, suggesting that the targeted drug-loaded liposomes may possess superior tumor targeting capability, enabling greater accumulation in tumor tissue within 4 h. After 12 h, fluorescence signals at tumor sites remained elevated in both groups, particularly in the Dir/Cu-Cur DTLPs group where fluorescence intensity remained notably prominent, further demonstrating the prolonged retention capability of Cu-Cur DTLPs at tumor sites. After 24 h, fluorescence signals at tumor sites decreased in all three groups. However, the Dir/Cu-Cur DTLPs group maintained relatively high fluorescence intensity at the tumor site, indicating that the targeted drug-loaded liposomes accumulated more persistently at the tumor site over an extended period compared to the other two formulations.

In the fluorescence distribution results across various visceral regions: The Dir solution group exhibited distinct fluorescence signals in the liver and spleen regions after 1 h, excluding the tumor site, indicating rapid uptake of the Dir solution by the mononuclear-macrophage system. Fluorescence in the kidney region was weaker, possibly due to Dir solution passing through the kidneys via the bloodstream. After 4 h, fluorescence intensity increased in the liver and spleen regions, while the kidney region showed a slight increase in fluorescence signal. Weak fluorescence began to appear in the intestinal tract, likely due to partial excretion of Dir solution after hepatic metabolism. After 12 h, fluorescence diminished in the liver and spleen regions, remained largely stable in the kidney region, and increased in the intestinal tract. After 24 h, fluorescence in the liver and spleen regions decreased significantly, fluorescence in the kidney region declined, while the intestine region maintained a certain level of fluorescence.

The Dir/Cu-Cur LPs group exhibited strong fluorescence in the liver and spleen regions after 1 h, likely due to rapid recognition of the liposomes by the mononuclear-phagocytic system. After 4 h, fluorescence in the liver and spleen regions further intensified, with enhanced fluorescence signals in the kidney region and the emergence of faint fluorescence in the intestine. After 12 h, fluorescence in the liver and spleen regions remained at high levels, fluorescence in the kidney region was largely stable, and fluorescence in the intestinal region increased. After 24 h, fluorescence in the liver and spleen regions decreased but remained at relatively high levels, fluorescence in the kidney region decreased, and fluorescence in the intestinal region remained quite noticeable.

The Dir/Cu-Cur DTLPs group exhibited weaker fluorescence in the liver and spleen regions after 1 h, likely due to slower recognition of the targeted liposomes by the mononuclear-macrophage system. After 4 h, fluorescence in the liver and spleen regions increased but remained lower than that in the Dir/Cu-Cur LPs group. Fluorescence signals in the kidney region intensified, and faint fluorescence began to appear in the intestine. After 12 h, fluorescence in the liver and spleen regions remained at a certain level, fluorescence in the kidney region was largely stable, and fluorescence in the intestinal region increased. After 24 h, fluorescence in the liver and spleen regions decreased, fluorescence in the kidney region decreased, and fluorescence in the intestinal region still showed some presence.

Overall, the targeted liposomes (Dir/Cu-Cur DTLPs group) exhibited stronger fluorescence signals at the tumor site, indicating superior targeting and more efficient delivery of the curcumin–copper chelate to tumor tissues. Dir solution demonstrated rapid in vivo distribution and metabolism, whereas both liposomal formulations, due to the protective effect of the liposomes, persisted in vivo for a relatively extended period and continuously distributed to the tumor site. Fluorescence distribution in visceral regions across all time points for the three groups reflected distinct characteristics in distribution, metabolism, and excretion among formulations. Targeted drug-loaded liposomes significantly reduced non-specific uptake in non-tumor tissue.

**Figure 8:**
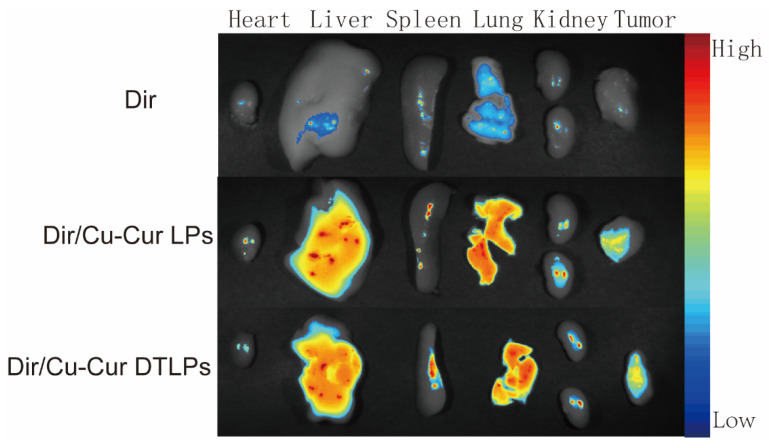
Fluorescence distribution in excised major organs and tumor tissues of tumor-bearing mice 24 h after injection of the formulation.

**Figure 9:**
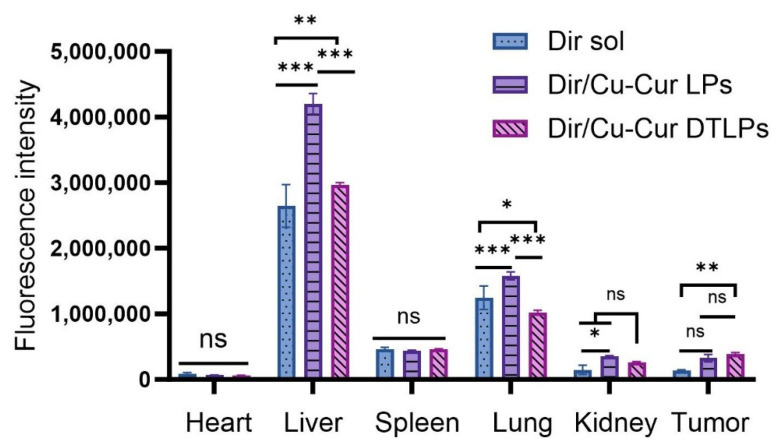
Fluorescence Intensity of Isolated Tissue. Data are shown as mean ± SD. One way ANOVA with Tukey’s multiple comparisons test. ns *p* > 0.05, * *p* < 0.05, ** *p* < 0.01, *** *p* < 0.001.

In fluorescence distribution experiments on excised mouse organs and tumor tissues (Figure 8 and Figure 9), after 24 h, the Dir solution group exhibited relatively low overall fluorescence intensity across all organs and tumor tissues, predominantly appearing blue or light blue. This may be attributed to its metabolism by the liver and kidneys, as well as its gradual distribution throughout various tissues, organs, or bodily fluids via blood and fluid circulation, thereby reducing its concentration and fluorescence intensity at specific sites.

The Dir/Cu-Cur LPs group exhibited higher fluorescence intensity in organs such as the liver, spleen, and lungs, appearing yellow-orange or even red, indicating that conventional liposomes enable effective distribution and enrichment of Dir in these organs. As a key organ for metabolism and detoxification, the liver likely exhibits uptake and metabolic processing of the liposomes. The abundant reticuloendothelial system cells in the spleen and lungs may have engulfed the liposomes, resulting in elevated fluorescence signals. In contrast, fluorescence intensity in the heart and kidneys was relatively low, appearing predominantly light blue to blue.

The Dir/Cu-Cur DTLPs group also exhibited high fluorescence intensity in the liver, potentially related to lipid body uptake by the liver and interactions between certain components of the dual-targeted liposomes and liver cells. In other organs such as the spleen, lungs, heart, and kidneys, fluorescence intensity was relatively moderate, primarily appearing yellow to light blue. This indicates relatively balanced distribution of the Dir/Cu-Cur DTLPs formulation in these organs. However, compared to the Dir/Cu-Cur LPs group, fluorescence intensity in the spleen and lungs was less pronounced. This may result from the dual-targeting mechanism favoring tumor tissue targeting, thereby reducing non-specific accumulation in certain normal organs.

In tumor tissues, the Dir/Cu-Cur LPs group exhibited distinct fluorescent signals, predominantly yellow to orange, indicating that conventional liposomes can deliver Dir to tumor tissues with some degree of tumor targeting. However, compared to the Dir/Cu-Cur DTLPs group, their fluorescence intensity was relatively weaker. The Dir/Cu-Cur DTLPs group exhibited higher fluorescence intensity in tumor tissue than the Dir/Cu-Cur LPs group, displaying strong red fluorescence. This may be attributed to the dual-targeting mechanism of folate and mitochondria on the surface of the liposomes. In which folate specifically binds to folate receptors overexpressed on tumor cell surfaces, while the mitochondrial targeting component further facilitates liposome entry into tumor cell mitochondria. This enhances Dir accumulation efficiency within tumor tissue, resulting in the strongest fluorescence signal in the Dir/Cu-Cur DTLPs group and demonstrating superior tumor targeting.

### 2.5. In Vivo Antitumor Studies of Targeted Drug-Loaded Liposomes

#### 2.5.1. Results of Mouse Body Condition and Weight Changes

Following tumor cell inoculation, during the initial modeling phase (days 1–3), no palpable mass is visible in the right axillary region of the mouse, and no significant change in body weight is observed. During the growth phase (days 4–10), the tumor gradually enlarges, and a distinct mass becomes palpable.

Day 0 was designated as the first day of administration. At the onset of treatment, behavioral differences among mice in each group were minimal. After three administrations, mice in the PTX group exhibited increased irritability and displayed significant stress responses during dosing. Mice in the Cur sol group showed decreased alertness, dulled coat color, and reduced activity. Tumor-bearing mice in other formulation groups maintained good condition, exhibited increased activity, and showed no reduction in water intake.

**Figure 10:**
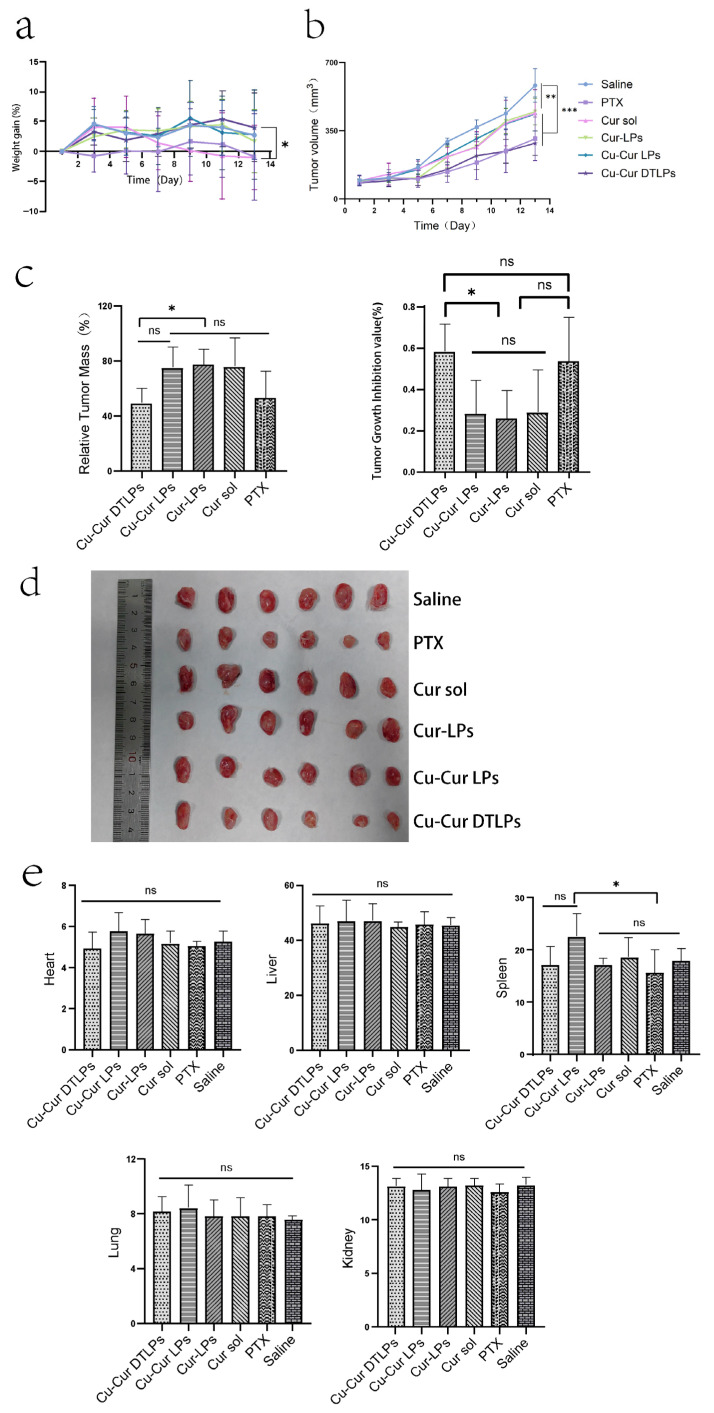
(**a**) Weight gain Over Time. Data are shown as mean ± SD. (**b**) Tumor Volume Changes Over Time in Mice of Each Group Following Drug Administration. Data are shown as mean ± SD. One way ANOVA with Tukey’s multiple comparisons test. (**c**) Relative Tumor Mass and Tumor Growth Inhibition Rate (TGI) in Mice of Different Groups. Data are shown as mean ± SD. One way ANOVA with Tukey’s multiple comparisons test. (**d**) In vitro mouse tumor tissue after 12 days of administration (*n* = 6). (**e**) Organ Indexes in Mice from Different Formulation Groups After 12 Days of Administration. Data are shown as mean ± SD. One way ANOVA with Tukey’s multiple comparisons test. ns *p* > 0.05, * *p* < 0.05, ** *p* < 0.01, *** *p* < 0.001.

Figure 10a shows the weight gain over time in mice after administration. The graph indicates that within the two weeks following tumor modeling and drug administration, the weight changes in mice across experimental groups exhibited distinct fluctuating trends. No consistent upward or downward trajectory was observed, with variations fluctuating between −10% and 10%.

The Saline group exhibited relatively stable weight changes, showing some fluctuations but maintaining an overall gradual trend. This indicates that without drug intervention, tumor modeling had a minimal impact on mouse weight, with variations primarily driven by normal physiological states and the mild effects of modeling itself. This provides a baseline reference for weight changes in other treatment groups. The PTX group exhibited slow weight gain initially, even experiencing negative growth. While weight recovered somewhat during the middle to late observation period, the overall increase remained smaller than that of the Saline group. This may be attributed to the toxic side effects of paclitaxel, a chemotherapy drug, which impacted the mice’s physiological functions and inhibited weight gain. This reflects the potential adverse effects associated with paclitaxel during tumor treatment. The Cur sol group exhibited significant fluctuations in body weight. These large variations may stem from the administration method of the curcumin solution, which could have caused unstable metabolic or action processes within the mice. This instability likely delivered a substantial short-term impact on the mice’s physiological state, consequently affecting their body weight.

The Cur-LPs group exhibited a gradual upward trend in body weight, showing fluctuations but remaining relatively stable overall. This indicates that after being encapsulated into liposomes, curcumin exerts a relatively mild and stable effect within the mice, reducing adverse stimulation to their bodies and thereby enabling more stable weight gain. Similarly to the Cur-LPs group, the Cu-Cur LPs group exhibited relatively stable weight changes with an upward trend and smaller fluctuations. This suggests that the addition of copper ions did not significantly adversely affect mouse weight, or that copper ions and curcumin may exhibit synergistic effects, influencing the physiological state of mice in a relatively stable manner. The Cu-Cur DTLPs group initially showed moderate weight gain, followed by gradually increasing fluctuations and later decelerated growth. This indicates minimal initial impact on body weight but suggests that over time, the targeted delivery system may alter drug distribution and action mechanisms within the body, subsequently affecting weight. Alternatively, long-term administration may have caused cumulative drug effects, leading to altered weight trends. Overall, the Cu-Cur DTLPs group exhibited the smallest body weight changes, indicating the lowest toxicity and potentially the most favorable therapeutic effect.

#### 2.5.2. Evaluation of Tumor Growth Inhibition Effect

The tumor volume changes in mice after administration show that tumor volume in the Saline group increased continuously with the number of days of administration. In contrast, tumor volume growth in the PTX group was relatively slower and significantly lower than that in the Saline group. The tumor growth rates of the curcumin-based formulations fell between those of the Saline and PTX groups. Notably, the Cu-Cur DTLPs group exhibited a relatively slower growth rate in the later stages, indicating that curcumin formulations inhibit tumor growth, with Cu-Cur DTLPs potentially demonstrating superior efficacy. In the relative tumor mass graphs for different groups: the Cu-Cur DTLPs group exhibited the lowest relative tumor mass, showing significant differences compared to other curcumin-related groups (Cu-Cur LPs, Cur-LPs, Cur sol) and the PTX group. This further indicates that Cu-Cur DTLPs performed best in reducing tumor mass, demonstrating significant tumor growth inhibition. Tumor growth inhibition rate plots across groups revealed higher suppression rates in the Cu-Cur DTLPs (approximately 60%) and PTX (approximately 55%) groups, significantly exceeding other curcumin-related formulations. Ex vivo tumor tissue images from mice 12 days post-administration visually demonstrate that the Saline group exhibited larger tumor volumes, while the PTX and curcumin formulation groups showed relatively smaller tumor volumes. Among these, the Cu-Cur DTLPs group displayed visually significantly smaller tumor tissue compared to other groups, consistent with the aforementioned tumor volume, relative tumor mass, and tumor inhibition rate results.

These results indicate that both paclitaxel and curcumin-related formulations exhibit certain inhibitory effects on tumor growth in mice. Among them, Cu-Cur DTLPs demonstrated outstanding performance in reducing tumor volume and mass while enhancing tumor inhibition rates, outperforming other curcumin formulations. This suggests that Cu-Cur DTLPs represent a highly promising drug delivery system for antitumor agents.

Note: (I denotes the Saline group; II denotes the PTX group; III denotes the Cur sol group; IV denotes the Cur-LPs group; V denotes the Cu-Cur LPs group; VI denotes the Cu-Cur DTLPs group)

#### 2.5.3. Safety Evaluation Results

##### Organ Index

As shown in Figure 10e, based on the organ index results from different mouse organs, after 12 days of administration, the paclitaxel (PTX) group and curcumin-related formulation groups (Cu-Cur DTLPs, Cu-Cur LPs, Cur-LPs, Cur sol) did not produce significant adverse effects on vital organs such as the heart, liver, spleen, lungs, and kidneys. Organ indices remained largely stable, indicating that these drugs possess a certain degree of safety within the experimental dosage and cycle.

##### Liver and Kidney Function Test Indicators

**Figure 11:**
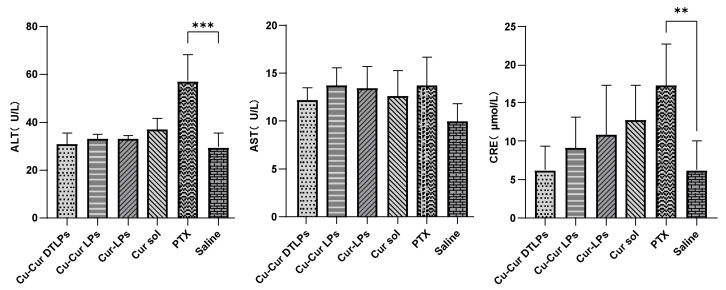
Key Indicators of Liver and Kidney Function in Serum of Mice from Each Group. Data are shown as mean ± SD. One way ANOVA with Tukey’s multiple comparisons test. ** *p* < 0.01, *** *p* < 0.001.

As shown in Figure 11, the ALT levels in the Cu-Cur DTLPs, Cu-Cur LPs, Cur-LPs, and Cur sol groups were relatively similar and showed no significant difference compared to the Saline group. This indicates that these curcumin-related formulations had no apparent effect on ALT levels in mouse livers under experimental conditions and did not cause significant liver damage. The PTX group exhibited significantly higher ALT levels than the Saline group, suggesting that paclitaxel may have caused some degree of liver damage in mice, leading to increased ALT release into the bloodstream and elevated ALT levels.

In the “AST (U/L)” graph, the AST levels across all treatment groups (Cu-Cur DTLPs, Cu-Cur LPs, Cur-LPs, Cur sol, PTX) showed no significant differences compared to the Saline group, with values remaining relatively close. This suggests that under the current experimental conditions, the drugs exerted minimal effects on AST levels in mouse livers and did not induce noticeable AST changes associated with liver injury.

The CRE (μmol/L) graph indicates that the CRE levels in the Cu-Cur DTLPs group were relatively low and showed no significant difference compared to the Saline group, suggesting that this formulation may not have a significant adverse effect on mouse kidney function. The CRE levels in the Cu-Cur LPs, Cur-LPs, and Cur sol groups also showed no significant difference from the Saline group, indicating that these curcumin formulations did not cause apparent renal impairment in mice during the experiment. The PTX group exhibited significantly higher CRE levels than the Saline group, suggesting that paclitaxel may have caused some renal damage in mice, leading to elevated creatinine levels and reflecting potential impairment of renal function. Overall, the different curcumin formulations showed no significant differences in their effects on serum factors, demonstrating good liver and kidney safety. However, based on numerical values, Cu-Cur DTLPs exhibited relatively superior performance in the kidney-related indicator CRE, potentially indicating a certain advantage in renal safety.

##### Complete Blood Count Results

**Figure 12:**
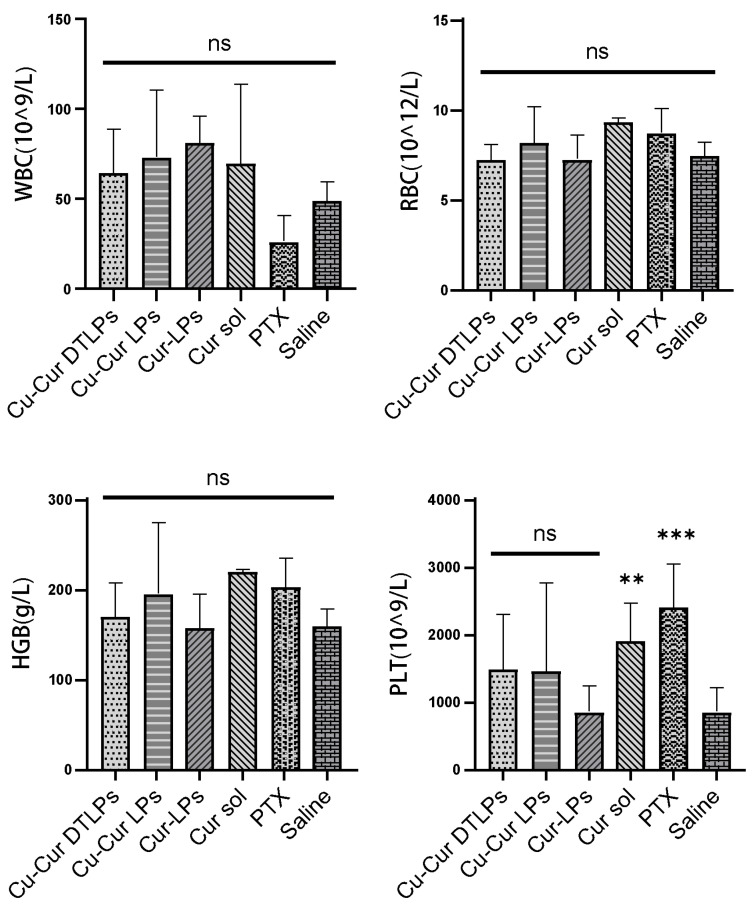
Complete Blood Count Results. Data are shown as mean ± SD (*n* = 3). One way ANOVA with Tukey’s multiple comparisons test. ns *p* > 0.05, ** *p* < 0.01, *** *p* < 0.001.

Figure 12 illustrate the blood count parameters in mice across different treatment groups, including white blood cell count (WBC), red blood cell count (RBC), hemoglobin (HGB), and platelet count (PLT). No significant differences in WBC, RBC, and HGB levels were observed between any treatment group (Cu-Cur DTLPs, Cu-Cur LPs, Cur-LPs, Cur sol, PTX) and the control group (Saline). This indicates that the experimental doses and cycles did not cause significant suppression of the immune system or leukopenia in mice, demonstrating favorable safety profiles. RBC and HGB levels showed no significant differences among all treatment groups and were consistent with the control group. This indicates that the different treatment groups did not cause anemia or erythropoietic dysfunction, suggesting no significant negative impact on bone marrow hematopoietic function. The Cu-Cur DTLPs group demonstrated superior safety in blood routine parameters, particularly with significantly higher PLT levels than other groups, potentially reflecting advantages in improving coagulation function or reducing toxic side effects. Compared to paclitaxel, curcumin-related formulations generally exerted lesser effects on the immune system and hematopoietic function. The Cu-Cur DTLPs group, in particular, demonstrated superior biocompatibility and safety. These findings further support Cu-Cur DTLPs as a promising antitumor therapeutic strategy with favorable safety profiles.

##### H&E Staining Results of Major Organs

**Figure 13:**
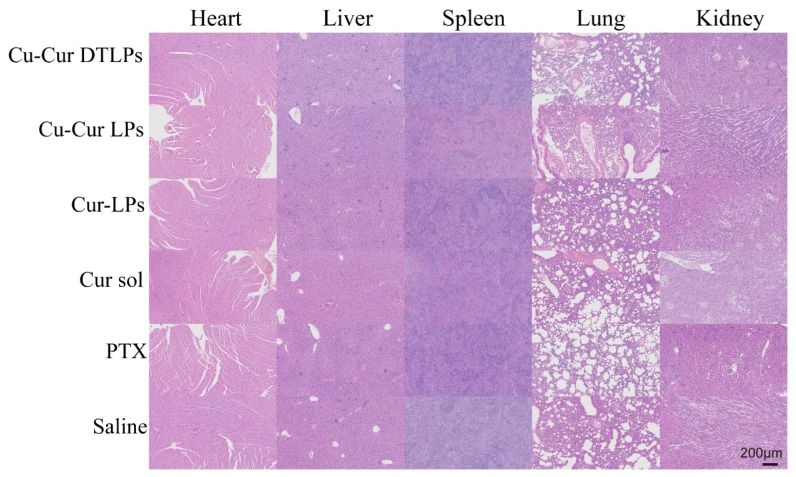
H&E staining results of major organs.

Histological analysis via H&E staining revealed minimal tissue damage in the Cu-Cur DTLPs group, indicating superior safety. Results for each group are shown in Figure 13: In cardiac tissue, the PTX group exhibited myocardial cell vacuolation and fiber rupture, consistent with its cardiotoxic profile. Other groups (including Cu-Cur DTLPs and Cu-Cur LPs) demonstrated well-organized myocardial cell arrangement without significant pathological changes. In liver tissue, hepatocytes in the Cu-Cur DTLPs group exhibited regular arrangement without significant necrosis or inflammation. The Cu-Cur LPs and Cur-LPs groups showed mild punctate necrosis, while the Cur sol group demonstrated more pronounced punctate necrosis and inflammatory response. The PTX group exhibited eosinophilic changes in hepatocytes and bile duct epithelial cell hyperplasia, consistent with its hepatotoxicity characteristics. The Saline group showed normal hepatocyte morphology. In spleen tissue, except for the PTX group, splenocytes in other groups exhibited clear structures without significant inflammation or necrosis. The PTX group showed lymphocyte depletion and splenocyte structural disruption, suggesting potential effects on immune organs. In lung tissue, alveolar structures remained largely normal in the Cu-Cur DTLPs group. The Cu-Cur LPs and Cur-LPs groups showed mild alveolar septal thickening, possibly indicating minor inflammatory responses, while the Cur sol group exhibited inflammatory cell infiltration. The PTX group demonstrated mild alveolar septal thickening without significant fibrosis or severe inflammation, and the Saline group showed normal tissue. In renal tissue, tubules and glomeruli appeared morphologically normal without significant damage in the Cu-Cur DTLPs group. The Cu-Cur LPs group exhibited mild tubular injury without obvious pathology. The Cur-LPs and Cur sol groups showed more pronounced tubular damage, suggesting potential effects of curcumin on renal function. The PTX group demonstrated mild injury but overall normal morphology, while the Saline group maintained normal morphology.

#### 2.5.4. Results of Antitumor Mechanisms

##### Results of Copper Ion Accumulation

**Figure 14:**
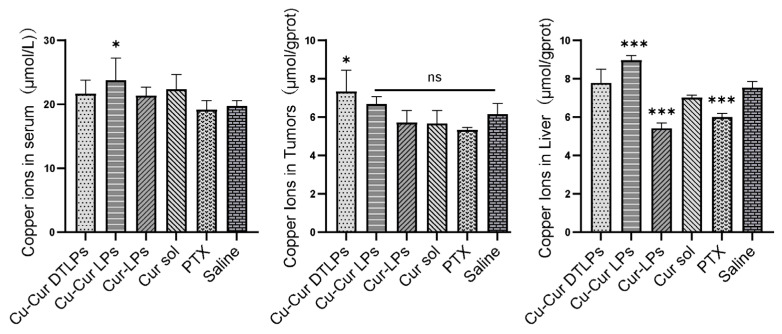
Copper Ion Levels in Serum, Tumor Tissue, and Liver Tissue Across Different Groups. Data are shown as mean ± SD. One way ANOVA with Tukey’s multiple comparisons test. ns *p* > 0.05, * *p* < 0.05, *** *p* < 0.001.

As shown in Figure 14, the Cu-Cur DTLPs group exhibited the highest copper accumulation in tumor tissue, while the Cu-Cur LPs group showed the highest copper accumulation in liver tissue and serum. This indicates that under the action of the targeting ligand, Cu-Cur is specifically delivered to the tumor site with minimal accumulation elsewhere, facilitating the activation of copper-induced cell death. Significant differences in copper ion levels were observed between the Cur-LPs group and the Cur sol group in liver tissue. This suggests that the liposome, as a drug carrier, protects curcumin from the effects of the internal environment, reducing the likelihood of rapid metabolism or degradation in organs such as the liver and enabling the slow release of curcumin. Under these conditions, curcumin can exert its effects continuously at relatively stable concentrations. This may exert a relatively mild yet persistent influence on copper ion transport, metabolism-related proteins, or channels within hepatocytes. Consequently, copper ion levels may exhibit specific trends, such as alterations in distribution potentially mediated through mechanisms like metabolic regulation or antioxidant effects, leading to reduced copper ion levels in liver tissue. Paclitaxel primarily targets tubulin in cells, inhibiting microtubule depolymerization and thereby disrupting mitosis. When exposed to paclitaxel, liver cells may experience impaired normal physiological functions and metabolic processes. This could indirectly affect proteins or organelles involved in copper ion uptake, transport, or storage, potentially reducing copper ion uptake or increasing its efflux from liver cells. Consequently, copper ion levels in liver tissue may decrease.

##### Analysis of IHC Staining Results

**Figure 15:**
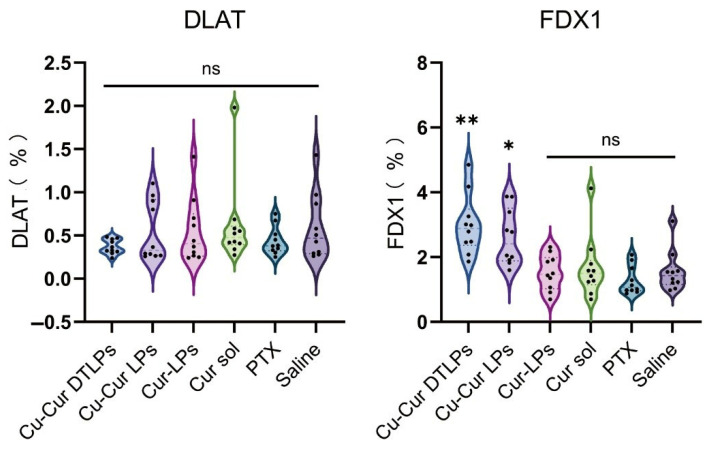
Percentage of cells expressing DLAT and FDX1. One way ANOVA with Tukey’s multiple comparisons test. ns *p* > 0.05, * *p* < 0.05, ** *p* < 0.01.

**Figure 16:**
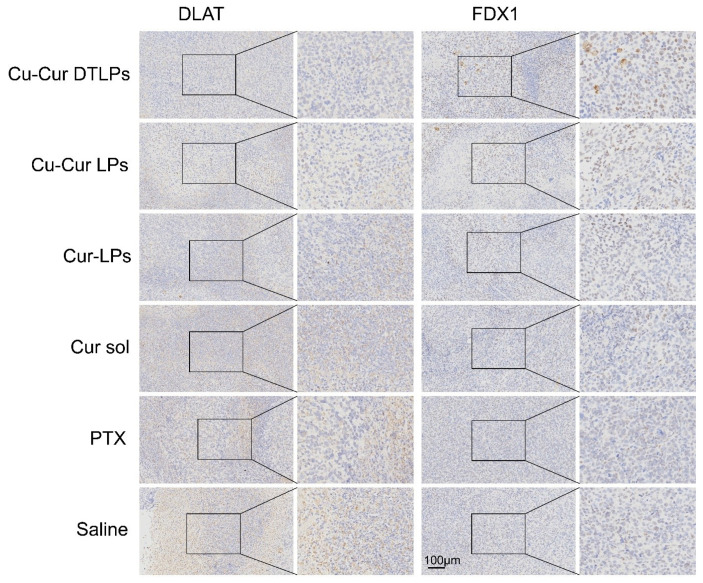
IHC staining results.

Immunohistochemical analysis revealed no statistically significant differences in the percentage of DLAT-positive cell area among groups. However, the Cu-Cur DTLPs group exhibited the lowest DLAT-positive area percentage (0.27 ± 0.08%), showing a decreasing trend compared to the saline group (0.59 ± 0.38%) (Figure 15). The IHC images in Figure 16 also visually demonstrate that the Cu-Cur DTLPs group exhibited less brownish-yellow material compared to the saline group, suggesting that Cu-Cur DTLPs may interfere with the function of the pyruvate dehydrogenase complex through mitochondrial-targeted delivery. In contrast, analysis of FDX1-positive cell area proportion revealed significantly higher FDX1 expression in the Cu-Cur LPs group (2.61 ± 0.86%) and Cu-Cur DTLPs group (3.00 ± 0.92%) compared to the saline group (1.56 ± 0.63%). IHC images revealed diffuse weak FDX1 staining in tumor cells of the saline group, whereas FDX1 signals in the Cu-Cur DTLPs group were concentrated in mitochondria-enriched regions and exhibited granular strong staining. These findings suggest that Cu-Cur DTLPs specifically activate the FDX1-dependent copper death pathway via copper ion accumulation, potentially through Cu^2+^/FDX1 reduction-driven lipoylated protein toxic aggregation. The differential regulatory patterns of DLAT and FDX1 suggest functional complementarity in copper-induced cell death: FDX1 mediates copper ion toxicity effects, while DLAT downregulation may reflect mitochondrial metabolic homeostasis disruption.

##### Metabolite Level Assessment

**Figure 17:**
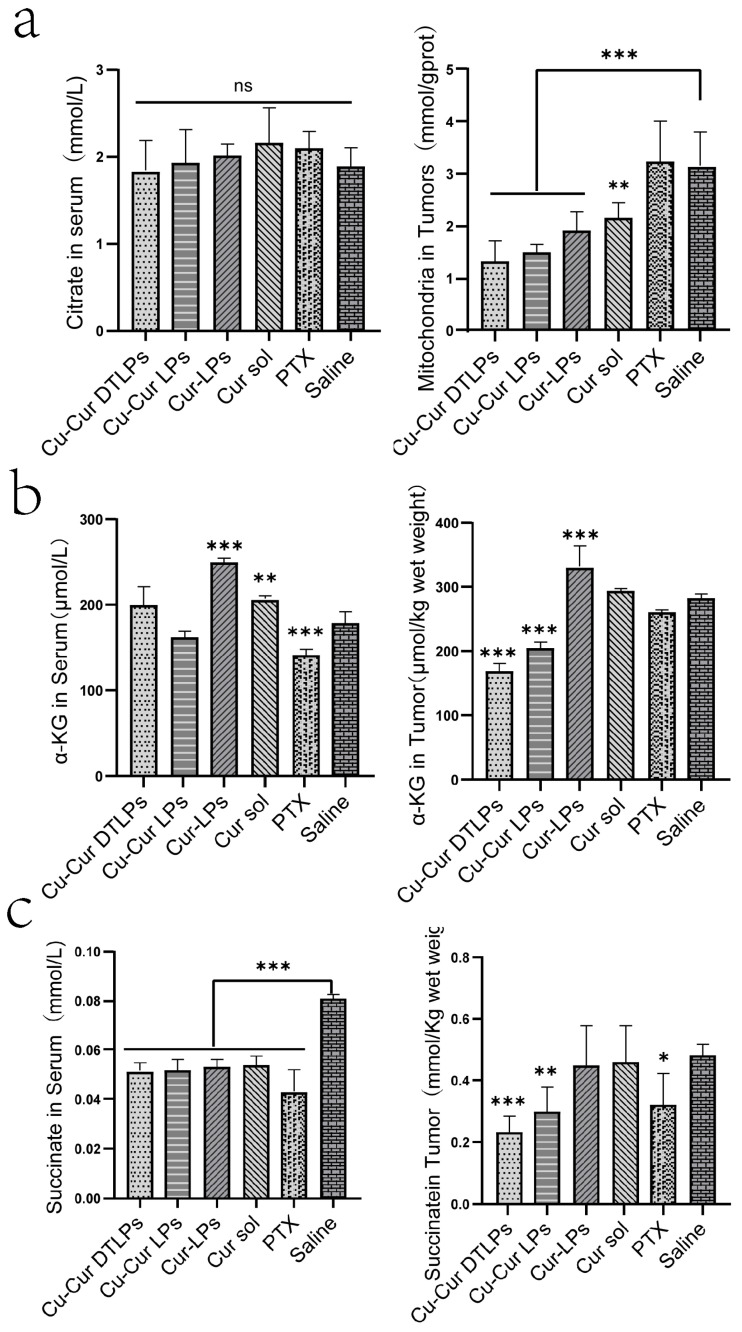
(**a**) Citrate Levels in Serum and Tumor Tissue Mitochondria. Data are shown as mean ± SD. One way ANOVA with Tukey’s multiple comparisons test. (**b**) Levels of α-Ketoglutarate (α-KG) in Serum and Tumor Tissue. Data are shown as mean ± SD. One way ANOVA with Tukey’s multiple comparisons test. (**c**) Succinate Levels in Serum and Tumor Tissue. Data are shown as mean ± SD. One way ANOVA with Tukey’s multiple comparisons test. ns *p* > 0.05, * *p* < 0.05, ** *p* < 0.01, *** *p* < 0.001.

Figure 17a displays citrate levels in serum and tumor tissue mitochondria across different subgroups. It shows no significant variation in serum citrate levels between subgroups, likely because serum citrate is influenced by multi-organ metabolism (e.g., liver, kidney, muscle), and localized changes in a single tumor tissue may be masked by systemic metabolic compensation. In tumor tissues, the citrate levels in mitochondria from the copper-containing formulation groups showed a significant decrease. Compared to the saline group, the reductions in Cu-Cur DTLPs and Cu-Cur LPs were 58.2% (*p* < 0.001) and 52.1% (*p* < 0.001), respectively. suggesting copper ions inhibit oxalate synthase activity, blocking citrate conversion to isocitrate and halting the TCA cycle. The greater citrate reduction in the targeted group indicates targeted delivery may enhance copper-induced death efficiency. The reduction in citrate levels within tumor tissue, coupled with no significant changes in serum levels, partially supports the copper-induced death mechanism. This suggests that the TCA cycle is specifically inhibited by copper ions locally within the tumor, while serum levels are maintained by metabolic processes in other organs.

Alpha-ketoglutarate levels in tumor tissues and serum, as shown in Figure 17b, decreased in both the Cu-Cur DTLPs group and the Cu-Cur LPs group. From the perspective of copper death mechanisms, copper-induced cell death disrupts the mitochondrial respiratory chain and iron–sulfur clusters, affecting the tricarboxylic acid cycle (in which α-ketoglutarate participates), leading to reduced synthesis or increased consumption. This result aligns with the expectation that copper-induced cell death alters α-ketoglutarate levels through metabolic effects, suggesting potential copper-induced cell death. The α-ketoglutarate levels in the Cur-LPs and Cur sol groups did not show a significant decrease comparable to that in the Cu-Cur DTLPs group. This indicates that pure curcumin affects α-ketoglutarate metabolism in tumor cells differently from curcumin–copper chelates, indirectly suggesting that copper ions chelated with curcumin produce new effects potentially related to copper death. Serum α-ketoglutarate levels showed no significant difference between the Cu-Cur groups and the Saline group. while Cur-LPs and Cur sol groups exhibited elevated α-ketoglutarate levels. This increase likely stems from curcumin regulating hepatic metabolic function to promote α-ketoglutarate synthesis and release into serum. The higher Cur-LPs values compared to Cur sol may result from the enhanced bioavailability of curcumin facilitated by the liposomal formulation.

Figure 17c demonstrates that serum succinate levels in the Cu-Cur DTLPs group, Cu-Cur LPs group, Cur-LPs group, and Cur sol group were relatively similar and lower than those in the saline group. Curcumin and its chelates may influence the metabolism of succinate in organs such as the liver by regulating the body’s metabolism, preventing serum succinate levels from exceeding those of the control group. The specific mechanism may involve promoting succinate metabolism or reducing its release from tissues into serum. The PTX group exhibited the lowest levels. Taxol’s interference with metabolic processes may have disrupted hepatic synthesis, metabolism, and release of succinate, leading to markedly reduced serum succinate levels. In tumor tissues, the Cu-Cur DTLPs group exhibited the lowest succinate levels. From the perspective of copper death mechanisms, Cu-Cur induces copper death by disrupting the mitochondrial respiratory chain and iron–sulfur clusters, thereby impairing the tricarboxylic acid (TCA) cycle. Succinate dehydrogenase contains iron–sulfur clusters, and its activity is inhibited. Although there may be a tendency for succinate accumulation, the severe disruption of the overall TCA cycle results in more pronounced inhibitory effects on succinate production at other stages, leading to the lowest levels among all groups. This partially aligns with the metabolic profile observed during copper death under severely disrupted TCA cycle conditions. The Cu-Cur LPs group, lacking targeted action, exerted relatively minor effects on the TCA cycle, resulting in higher succinate levels than the Cu-Cur DTLPs group. Nevertheless, its succinate levels remained lower than those observed in other formulation groups.

##### H&E Staining and TUNEL Staining Analysis

**Figure 18:**
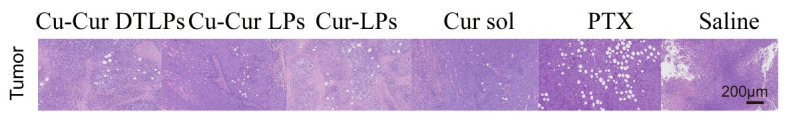
H&E staining results of tumor tissue (scale bar: 200 μm).

H&E staining of tumor tissues is shown in Figure 18. The Cu-Cur DTLPs group exhibited significantly reduced tumor cell density with extensive necrosis. The Cu-Cur LPs group showed decreased cell density but fewer necrotic areas. The Cur-LPs and Cur sol groups demonstrated weaker antitumor effects. The PTX group exhibited larger necrotic areas.

**Figure 19:**
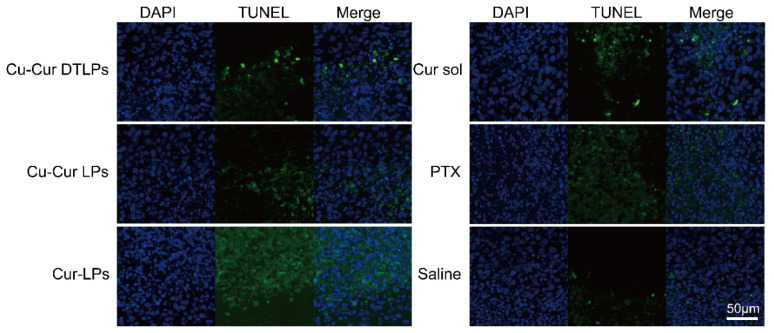
TUNEL staining results in tumor tissue (scale bar: 50 μm).

As shown in the TUNEL staining results for each group in Figure 19, the PTX group exhibited the highest level of tumor tissue apoptosis. The apoptosis level in liposomes prepared with the Cu-Cur complex was lower than that in liposomes prepared directly with curcumin or in curcumin solution. This indirectly suggests that the antitumor effect of Cu-Cur may primarily occur through copper-mediated death rather than apoptosis.

## 3. Discussion

This study focuses on the application of curcumin–copper chelate-targeted liposomes (Cu-Cur DTLPs) in breast cancer treatment, aiming to develop a safe and effective novel therapeutic strategy. Through a series of experiments, the antitumor efficacy and related mechanisms of Cu-Cur DTLPs were successfully validated both in vivo and in vitro, providing new insights and potential approaches for breast cancer treatment.

In the research design, based on the copper death mechanism and the synergistic effects of curcumin and copper, a curcumin–copper chelate (Cu-Cur) was constructed. Through a targeted liposome delivery system, copper accumulation at the tumor site was achieved to selectively induce copper death in tumor cells. The research process encompassed multiple critical stages: chelate synthesis and characterization, liposome preparation and evaluation, in vitro cell experiments, in vivo targeting studies, and in vivo antitumor experiments.

Regarding chelate synthesis and characterization, Cu-Cur was successfully synthesized. Its structure was comprehensively analyzed and validated using multiple detection techniques including mass spectrometry, NMR, thermogravimetry, UV, and IR spectroscopy. Results indicated a filtrate yield of 50–60%, yielding an orange-red crystalline powder, while the filter residue produced a reddish-brown powder. Comprehensive analysis of both products confirmed the structural characteristics, crystallinity, and composition of the reaction products, establishing a crucial foundation for subsequent studies.

In the liposome preparation and evaluation experiments, three types of liposomes—Cu-Cur DTLPs, Cu-Cur LPs, and Cur-LPs—were synthesized using the thin-film dispersion method. A UV spectrophotometric assay was established for content determination, demonstrating excellent precision and stability for both Cur and Cu-Cur under this method. Characterization parameters including particle size, polydispersity index (PDI), zeta potential, transmission electron microscopy morphology, stability, drug loading capacity, encapsulation efficiency, and in vitro release were measured. Results indicated that Cu-Cur DTLPs exhibited smaller particle size, more spherical morphology, and excellent stability. They demonstrated effective drug release in both tumor microenvironments and in vivo settings, exhibiting superior properties as drug delivery carriers.

In vitro cytotoxicity assessments using 4T-1 and MDA-MB-231 cells as models evaluated Cur- and Cu-Cur-loaded liposomes via MTT assays. Results demonstrated significant inhibitory effects of Cu-Cur LPs on both cell lines.

In vivo targeting studies employed a BALB/c female mouse 4T-1 breast cancer model. Lipid formulations labeled with DIR fluorescent dye were administered via IVIS small animal imaging systems to observe their distribution within mice and accumulation in tumor tissues. Results demonstrated that compared to the fluorescent dye solution and conventional liposomes, Cu-Cur DTLPs exhibited stronger fluorescence signals at tumor sites, enabling more efficient delivery of the curcumin–copper chelate to tumor tissues. This approach demonstrated superior tumor targeting and prolonged circulation, while also reducing non-specific uptake in non-tumor tissues to a certain extent.

In vivo antitumor experiments were also conducted using the 4T-1 breast cancer BALB/c mouse model to evaluate the antitumor efficacy of Cu-Cur DTLPs. Through monitoring tumor growth curves, observing changes in tumor volume and weight, conducting pathological analysis of vital organs and serum marker analysis, and employing relevant assay kits to investigate mechanisms, results demonstrated Cu-Cur DTLPs’ superior efficacy in reducing tumor volume and mass while enhancing tumor suppression rates compared to other curcumin formulations. Concurrently, Cu-Cur DTLPs exhibited minimal toxicity to major organs and demonstrated favorable safety. Its mechanism primarily involves inducing copper accumulation in tumor tissues, disrupting the TCA cycle within tumor cells, and consequently causing metabolic dysfunction and cell death.

In summary, this study successfully validated the in vivo and in vitro antitumor efficacy of Cu-Cur DTLPs in a mouse breast cancer model. The targeted delivery of curcumin–copper chelates offers a safe and effective approach for inducing copper death and inhibiting tumor growth. Cu-Cur DTLPs hold potential as a novel therapeutic strategy for breast cancer, promising new breakthroughs in clinical treatment. Future research should focus on elucidating the molecular mechanisms underlying Cu-Cur DTLPs-induced copper death, evaluating the efficacy of combining Cu-Cur DTLPs with other anticancer therapies, conducting pharmacokinetic studies, optimizing delivery regimens, and assessing the potential of Cu-Cur DTLPs for treating other types of cancer.

In the field of targeted therapy for triple-negative breast cancer (TNBC), nanoparticle delivery systems have emerged as a research hotspot due to their ability to enhance drug targeting efficiency and therapeutic specificity. Compared to other recently reported advanced nanoscale systems such as hyaluronic acid-targeted chemo-photothermal synergistic nanoparticles (HA-ANI P/I NPs) [43] and pH/GSH dual-responsive nanoparticles (ADR-Ola-NP) [44], Cu-Cur DTLPs reflect common trends in targeting strategies, therapeutic mechanisms, and formulation design while also demonstrating unique innovations. Regarding targeting strategies, these formulations all aim to enhance drug enrichment in tumor tissues. HA-ANI NPs utilize hyaluronic acid (HA) to target the CD44 receptor highly expressed on the surface of TNBC cells, achieving active targeting. Cu-Cur DTLPs similarly employ targeted liposome design, with in vivo imaging confirming preferential accumulation and enrichment at tumor sites—aligning with mainstream active targeting principles. Additionally, these formulations benefit from passive targeting via the enhanced penetration and retention (EPR) effect conferred by their nanoscale size. Furthermore, ADR-Ola-NP achieves intelligent responsiveness to the tumor microenvironment through sophisticated chemical design—such as pH-sensitive hydrazone bonds and GSH-sensitive disulfide bonds—enabling controlled drug release and further enhancing targeting efficiency. Regarding therapeutic mechanisms, Cu-Cur DTLPs innovatively combine the cutting-edge “copper death” mechanism with the natural compound curcumin, differing from other formulations that focus on combining known therapies. For instance, HA-ANI NPs integrate the chemotherapeutic agent paclitaxel (PTX) with the photothermal agent IR780, delivering synergistic chemothermo-therapeutic killing of tumor cells. ADR-Ola-NP co-loads doxorubicin (ADR) and the PARP inhibitor olaparib (Ola), achieving synergistic enhancement through a “synthetic lethality” effect that disrupts DNA synthesis while inhibiting repair. The core innovation lies in delivering a novel functional chelate, Cu-Cur, designed to attack tumor cells at the metabolic level. This achieves its goal by inducing copper-mediated cell death and synergizing with curcumin’s established multi-targeted antitumor activity, offering a novel pathway to overcome traditional drug resistance in TNBC. In formulation design, liposomes are considered carriers with promising clinical translation potential due to their excellent biocompatibility and relatively mature manufacturing processes. The selection of liposomes as the carrier for Cu-Cur DTLPs may offer advantages in biosafety compared to approaches using synthetic polymers like PLGA or inorganic materials.

The clinical translation of curcumin–copper chelate-targeted liposomes (Cu-Cur DTLPs) faces multiple challenges, primarily in process scale-up, immunogenicity, in vivo clearance, and quality control. During process scale-up, the laboratory-scale membrane dispersion method faces significant hurdles when scaled to industrial production. Minor variations in parameters such as film uniformity, hydration volume control, dispersion energy consumption, and thermal effects can lead to broadened particle size distribution index (PDI), reduced encapsulation efficiency, and batch-to-batch variability, severely compromising product consistency. Regarding immunogenicity, the formation of curcumin–copper chelates within the human body may generate novel antigenic epitopes, triggering specific immune responses. With repeated dosing, immune memory could accelerate blood clearance (ABC), diminishing therapeutic efficacy and increasing the risk of immune-related adverse events. During in vivo clearance, Cu-Cur DTLPs are readily recognized and phagocytosed by the mononuclear phagocytic system (MPS), including Kupffer cells in the liver and splenic macrophages. This leads to rapid decline in blood concentrations and reduced tumor targeting. Additionally, vascular endothelial cell surface receptors may mediate liposome adhesion and endocytosis, further impairing tumor accumulation. Regarding raw materials and quality control, batch-to-batch variations in phospholipids, cholesterol, and other ingredients (e.g., purity, oxidation levels) directly impact liposome stability and drug loading capacity. Establishing stringent raw material quality standards and process parameters suitable for large-scale production are critical to ensuring product quality. Therefore, to advance the clinical translation of Cu-Cur DTLPs, systematic optimization of the liposome formulation and preparation process is required to reduce immunogenicity and improve long-circulation performance. Concurrently, a robust large-scale production and quality control system must be established.

## 4. Materials and Methods

### 4.1. Materials

Curcumin (Cur) and Copper acetate (Cu(AcO)_2_ were obtained from Shanghai Sigma-Aldrich Trading Co., Ltd. (Shanghai, China). Lecithin, DSPE-MPEG2000 and Egg phosphatidylglycerol (EPG) were acquired from Shanghai AVT Pharmaceutical Technology Co., Ltd. (Shanghai, China) Cholesterol (Cho) was sourced from Beijing Coolaber Technology Co., Ltd. (Beijing, China). DSPE-mPEG2000-FA was obtained from Xi’an Ruixi Biological Technology Co., Ltd. (Xi’an, China). DSPE-mPEG2000-MLS was purchased from Suzhou Qiangyao Biotechnology Co., Ltd. (Suzhou, China). Trichloromethane and Methanol were purchased from Tianjin Kangke Technology Co., Ltd. (Tianjin, China). Twain-80 was obtained from Sichuan Jinshan Pharmaceutical Co., Ltd. (Meishan, Sichuan, China). Dextran Gel G-50 and Thiazole blue (MTT) were obtained from Beijing Solarbio Science & Technology Co., Ltd. (Beijing, China). Fetal Bovine Serum (FBS) was obtained from Israel’s Boehringer-Ingelheim Company (Rhineland-Palatinate, Germany). DIR iodide was purchased from Dalian Meilun Biotechnology Co., Ltd. (Dalian, China). Paclitaxel was purchased from Xi’an Jinheng Chemical Co., Ltd. (Xi’an, China). Citric Acid Colorimetric Test Kit, Aspartate aminotransferase colorimetric test kit, Alanine aminotransferase colorimetric test kit, Creatinine colorimetric test kit, Succinic acid colorimetric test kit and Amplex Red α-Ketoglutarate Assay Kit were purchased from Wuhan Elabscience Biotechnology Co., Ltd. (Wuhan, China).

### 4.2. Animals

Healthy BALB/c female mice, SPF grade, body weight (18 ± 2) g, Speifu (Beijing) Biotechnology Co., Ltd. (Beijing, China) Animal room temperature: (25 ± 2) °C, relative humidity: (50 ± 2)%. The relevant research was conducted in accordance with the principles of animal experimentation and supported by the Animal Research Center of Tianjin University of Traditional Chinese Medicine, and the experimental operation process was recognized by the Animal Ethics Committee of Tianjin University of Traditional Chinese Medicine (ethics number: TCM-LAEC2024139g1105).

### 4.3. Methods

#### 4.3.1. Chemical Synthesis of Cu-Cur

**Figure 20:**
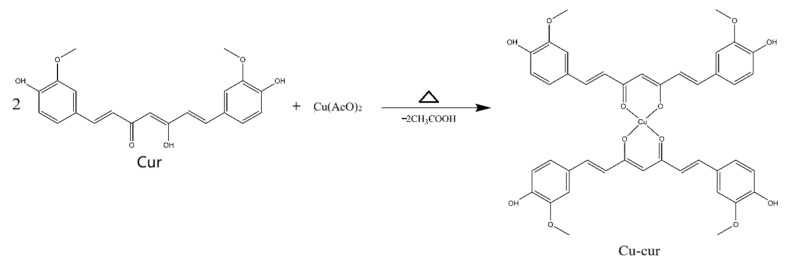
Cu-Cur Synthesis Route.

A: 200 mg curcumin mixed with 30 mL methanol, B: 50 mg copper acetate (Cu(AcO)_2_) mixed with 50 mL methanol(pH = 6.5), A and B dissolved at 60 °C, respectively. Add A droplets to B solution and stir the reaction for 12 h to obtain a mixed solution. The solution obtained after the reaction is filtered, the filter residue is placed in a constant temperature drying oven for vacuum drying (25 °C), the filtrate is transferred to a 40 °C water bath to slowly evaporate the solvent, and the resulting solids are repeatedly washed by deionized water under ice bath conditions, and finally dried in a vacuum environment (25 °C), and the crystalline solids obtained are Cu-Cur. (All steps except those performed under vacuum conditions are conducted in air). The synthetic route is shown in Figure 20.

#### 4.3.2. Structural Identification of Cu-Cur

1. Determination of electron paramagnetic resonance (EPR) results of filtrate products and filter residue products

Directly weigh 10 mg of powder sample into the sample tube, put the sample tube into the machine to test and collect data, test at low temperature 77 K to obtain the EPR results of the filtrate product and compare and analyze the EPR map of the filter residue product obtained after the reaction.

2. X-ray diffraction (XRD) determination of filtrate products and residue products

After grinding the sample, the instrument tube current was set to 40 mA, tube voltage 40 kV, and the Cu target wavelength was 1.5406 Å. The scanning range was set to 3–70° and the scanning speed was 6°/min, and the XRD maps of filtrate products, residue products and curcumin were compared.

3. Ultraviolet spectroscopy of Cu-Cur

Weigh a certain amount of Cur and Cu-Cur and dilute them with methanol. The spectra of Cur and Cu-Cur under UV conditions were obtained by a UV spectrophotometer with a measurement range of 200–800 nm, and the UV spectral curves of the two were compared.

4. Analysis of infrared spectral information of Cu-Cur

Take an appropriate amount of Cu-Cur powder, Cur powder and potassium bromide (KBr) powder in the oven to dry and remove moisture, add the pharmaceutical powder and KBr powder in an agate mortar at a ratio of 1:150 times, and grind in the same direction with a mortar until the color is evenly mixed and there is no particulate matter. After taking an appropriate amount of mixed grinding powder and adding it to the tableting device, gently shake it to flatten the surface of the powder, and then place the tablet pressing device on the tablet press for pressure pressing. After measuring the blank background, the infrared spectrometer is put into the pressed drug-containing KBr sheet, and the measured spectral wavenumber is 4000–400 cm^−1^. The synthesis was confirmed by comparing the changes in characteristic peaks between different samples.

5. Nuclear magnetic spectroscopy (NMR) information analysis of Cu-Cur

Weigh about 20 mg of Cur powder, dried solids with residue products and dried solids with filtrate products, added to nuclear magnetic tubes, and then dissolved in deuterated chloroform (CDCl3), shake to make the samples completely dissolved and put into the instrument for testing, the resonance frequency of 1H was 600 MH_Z_, sampled 16 times, relaxation time was 2 s, and the NMR spectral information of the product was recorded and compared.

6. Mass spectrometry analysis of Cu-Cur

Cu-Cur powder was dissolved in methanol, diluted to 200–300 ng/mL, and detected using a triple quadrupole mass spectrometer, under the following conditions: electrospray (ESI) ion source, positive ion mode detection, scanning mode was full scan (*m*/*z*: 100–1500), capillary voltage: 4 kV, sprayer gas pressure: 15 psi; Dry gas flow: 11 L/min, gas temperature: 300 °C; Elution gradient: 5% B held for 0.5 min, rose to 95% B in 1 min, held at 95% B for 3.5 min, then adjusted to 5% B in 1 min, and stopped after 5% B held for 2 min (Phase A: 0.1% formic acid; Phase B: acetonitrile); Column: Waters Sun Fire C18 (50 ×4.6 mm, 5 μm); Injection volume 5 μL; flow rate 0.2 mL/min; Column temperature at room temperature. The actual relative molecular mass of Cu-Cur is summarized by the spectral peaks and compared with the theoretical relative molecular mass.

7. Determination of the melting point of Cu-Cur

After loading an appropriate amount of sample into the melting point tube, put it into the melting point meter, heat the instrument to observe the powder state, record its initial and final melting temperatures, and determine the melting point of Cur according to the same operation.

8. Thermogravimetric determination of Cu-Cur

The sample was properly ground and put into the sample table of the thermogravimetric analyzer, adjusted to the appropriate sample weight, set the heating rate of the instrument to 5 °C/min, and the temperature range was 25–800 °C, and the mass-temperature data of Cu-Cur was obtained, and the thermogravimetric curve (TGA curve) was drawn to compare and analyze with the TG curve of Cu.

9. Oil–water partition coefficient (logP) of Cur and Cu-Cur was determined

Determination of standard curves of Cur and Cu-Cur (dissolution of n-octanol): Cur: Take a certain amount of Cur and dilute it with n-octanol to obtain a standard solution with concentrations of 12.98, 9.74, 8.65, 4.33, 2.16, 0.04 μg/mL, and measure the absorbance with UV-spectrophotometer and draw it. Cu-Cur: Take a certain amount of Cu-Cur powder and dilute it with n-octanol to obtain a standard solution with concentrations of 13.75, 9.16, 4.58, 2.29, 0.458, 0.05 μg/mL, and also measure the absorbance and draw the map.

A certain amount of n-octanol reagent and ultrapure water were shaken on the shaker for 24 h to be pre-saturated, and the two phases were collected separately after standing stratification, weighing about 10 mg of Cu-Cur and the same quality of Cur and placing them in a 100 mL corked erlenic flask, adding a certain volume of water-saturated n-octanol to fully dissolve and then fixing the volume of 100 mL. A certain volume of water-saturated n-octanol drug solution was added to a 50 mL corked erlenic flask, and different volume ratios of n-octanol saturated with n-octanol and water-saturated n-octanol (1:2, 1:1, 2:1) were selected to test the partition coefficient of the samples, and three parallel samples were set in each group. The conical flask was placed in a constant temperature water bath with magnetic stirring function and stirred continuously for 24 h, so that the drug was fully dissolved in the two phases and reached equilibrium, and then transferred to a 100 mL separation funnel for static stratification to separate the two phases. The content of Cu-Cur or Cur in the oil phase and the aqueous phase diluted by a certain number was determined, respectively, and the Pow was calculated according to the following formula, and finally the oil–water partition coefficient was obtained by calculating lgPow, and the calculation formula was as follows:

Pow = Cn-o/Cw(3)

where Cn-o is the equilibrium concentration of the substance in n-octanol, and Cw is the equilibrium concentration in the aqueous substance, the unit is micrograms per milliliter (μg/mL).

#### 4.3.3. Preparation and Evaluation of Cu-Cur Dual-Targeted Liposomes

1. Preparation of Cu-Cur Dual-Targeted Liposome(Cu-Cur DTLPs), Cu-Cur Liposome(Cu-Cur LPs) and Cur Liposome(Cur-LPs)

Cu-Cur DTLPs were prepared by thin film dispersion method [26], and the following prescriptions were weighed proportionally: EPC:Cholesterol:EPG:DSPE-mPEG2k:DSPE-mPEG2k-FA:DSPE-mPEG2k-MLS:Cu-Cur = 120:20:20:56:2:2:4. The dissolved organic phase is mixed and poured into a round-bottom flask, and the organic phase is removed by rotational evaporation under the condition of vacuum extraction to form a thin and clear yellow film. The flask was placed in a constant temperature drying oven at 25 °C for one night under vacuum conditions, and on the next day, 5 mL of ultrapure water and an appropriate amount of small glass beads were added, the film was properly shaken to dissolve in ultrapure water, and then poured into the small beaker, and the lipid suspension was obtained by stirring on a magnetic stirrer at 400 rpm and 25 °C for 0.5 h. The lipid suspension was ultrasonically fragmented at 20% power for 2 min, and Cu-Cur DTLPs were obtained through a 0.22 μm filter membrane.

Cur-LPs are weighed according to the following prescription: EPC:Cholesterol:EPG:DSPE-mPEG2k:Cu-Cur/Cur = 120:20:20:60:4, and the preparation method is the same as Cu-Cur DTLPs.

2. Determination of particle size, polydispersity index (PDI) and zeta potential of drug-loaded liposomes

The prepared Cu-Cur DTLPs, Cu-Cur LPs and Cur-LPs were diluted with ultrapure water 10 times and then measured by Malvern particle size assay.

3. Transmission electron microscopy observation of drug-loaded liposomes in each group

Carefully remove the carbon-backed copper mesh with tweezers and place it in a Petri dish with tiled capping film for later use, ensuring that the copper mesh is free of dust, magazines, and other contaminants. Pipette to aspirate an appropriate amount of liposomal sample suspensions and gently add it to the carbon support membrane of the copper mesh with a drop volume of 10 μL to ensure that the sample is evenly covered on the surface of the copper mesh to avoid droplet accumulation or the absence of samples in the area. After letting the sample naturally adsorb on the copper mesh for 10 min, carefully suck off the excess sample suspensions with filter paper without touching the copper mesh, and use a pipette to suck an appropriate amount of 3% phosphotungstic acid solution droplets and add it to the copper mesh with liposome samples to ensure that the negative dye covers the sample area evenly, and the negative staining time is 3 min, and also carefully absorb the negative stain solution with filter paper. The copper mesh was dried and placed in a transmission electron microscope (TEM) for observation, and representative images were recorded.

4. Stability study of targeted drug-loaded liposomes

Store the prepared Cu-Cur DTLPs in a 4 °C refrigerator, with each batch consisting of three parallel samples. Sample each batch on days 1, 3, 5, 7, 14, and 28 to measure particle size, PDI, and potential. Simultaneously observe the liposome condition, noting whether the suspensions remains clear and transparent with visible Tyndall effect.

5. Determination of Drug Loading Capacity and Encapsulation Efficiency of Liposomes

The specific steps are as follows: take an appropriate amount of dextran gel Spedex-G50 dry powder and soak it in 50–60% ethanol for 24 h at room temperature, stir continuously to make it swell, filter and wash off the residual ethanol with ultrapure water. The washed gel was stirred and swollen again with ultrapure water for 24 h, then soaked in 0.2 M HCl for 12 h, stirred and filtered in the gap, washed to neutral, filled with fully swollen dextran gel in a 2.5 mL syringe barrel pre-loaded with filter paper, centrifuged at 1500 rpm for 3 min, and 0.5 mL of deionized water was added to equilibrate the gel column at the same time and speed. Add 0.5 mL of Cu-Cur DTLPs, Cu-Cur LPs and Cur-LPs suspensions to the top of the gel column, centrifuge at 1500 rpm for 3 min, and then elute twice with the same volume of deionized water, and the bottom of the centrifuge tube is fully encapsulated liposomal suspension. 1 mL of the prepared liposomal suspension and the separated liposomal suspension were taken from the same batch, and 3 times the volume of methanol was added to ultrasonic demulsification, and the content was determined according to the ultraviolet detection wavelength. The encapsulation efficiency (EE) and drug loading efficiency (DLE) of liposomes were calculated according to the following formula.

DLE(%) = W1/W × 100%(4)

EE(%) = W1/W2 × 100%(5)

W1 is the mass of the drug contained in the liposome, W2 is the actual amount of drug contained in the system, and W is the total mass of the added drug and membrane.

6. Investigation of in vitro release of drug-loaded liposomes

The release rate was determined by dialysis, and buffers containing pH 5.0 and pH 7.4 containing 1% Tween-80 were selected as the elution medium to simulate the tumor microenvironment and in vivo environment, respectively. 1 mL of the preparation was added to a dialysis bag of appropriate length, the mouth of the bag was tightened, and placed into a 100 mL conical flask containing the release medium. The container was placed in a transdermal diffusion tester, set to a temperature of 37 °C and a speed of 100 rpm, and after the start of the experiment, 3 mL of release medium was removed from the container at 0.5 h, 1 h, 2 h, 4 h, 6 h, 10 h, 12 h, 24 h, and 48 h, respectively, and then supplemented with the same volume of buffer at the same temperature. The concentration of drugs in the sample solution was determined by UV-Vis spectrophotometer. According to the measured absorbance results, the release amount of drugs at different times was calculated through the relevant standard curves, and the drug release curves were plotted, and each group of experiments was parallel three times.

The cumulative release percentage (%) of the drug at each time point was calculated using the following formula.

(6) $$\mathrm{The}\;\mathrm{cumulative}\;\mathrm{release}\;\mathrm{percentage}=\frac{C_{n}\;\times \;V1+V2\times \sum_{i=1}^{n-1}C_{i}}{M}\times 100\%$$

Among them, Cn is the drug concentration (μg/mL) measured at the nth sampling point. Ci is the mass concentration of the drug (μg/mL) measured at the i(n − 1) sampling point; M is the quality of the drug in the dialysis bag.

#### 4.3.4. In Vitro Cell Experimental Studies Targeting Drug-Loaded Liposomes

Cell culture: Using RPMI-1640 medium, 10% fetal bovine serum (FBS) and 1% penicillin-streptomycin (P/S) were added as antibiotics, the cell growth environment was 37 °C, 95% air and 5% carbon dioxide (CO_2_), the humidity in the incubator was maintained at 70–80%, and the cell density reached 80–90% for passage, usually with a passage ratio of 1:2 to 1:3, and 3 passages per week.

##### Evaluation of the Cytotoxic Effects of Liposomes In Vitro

1. Prepare MTT solution

PBS was mixed with MTT to 5 mg/mL, followed by microporous membrane (0.22 μm) to filter out bacteria or impurities, and stored at low temperature and away from light.

2. The proportion of viable cells was determined by MTT method

4T-1 cells and MDA-MB-231 cells were seeded in a 96-well plate, and the cells should be logarithmic growth cells, and the density should be adjusted to 8 × 10^3^ cells/well in advance. Add 100 μL of cell suspension to each well in a 96-well plate, fill the edge wells with PBS to reduce evaporation errors, and subsequently pre-incubate in a 37 °C, 5% CO_2_ incubator for 24 h to allow the cells to adhere to the wall. After the end of the culture, the culture medium should be aspirated, and 100 μL of curcumin solution (Cur sol), curcumin liposomes (Cur-LPs), curcumin–copper chelate (Cu-Cur), and curcumin–copper liposomes (Cu-Cur LPs) should be added according to the concentration gradient of 1, 2, 4, 8, 16, and 32 μg/mL, respectively. Incubation was aspirated for 24 h, 1 mL of MTT (diluted to 1 mg/mL with DMEM subculture, 0.22 μm filter sterilization) was added to continue the culture, and the culture was completed after 4 h. After carefully aspirating the culture medium, add 150 μL of DMSO and place it on a shaker at a constant temperature of 37 °C for 10 min to completely dissolve the methane crystals. The absorbance value of each well at OD = 490 nm was determined by a microplate reader, and the cell viability was calculated according to the following formula to calculate the effect of different preparations on the cells. Data analysis and plotting were performed using Graphpad Prism 10.1.2, and all results were expressed as mean (Mean) ± standard deviation (SD). Statistical significance was analyzed by significance *t*-test and one-way analysis of variance (ANOVA) (* represents *p* < 0.05, ** represents *p* < 0.01, *** represents *p* < 0.001).

(7) $$\mathrm{Cell}\;\mathrm{viability}\left(\%\right)=\frac{A_{s}-A_{b}}{A_{c}-A_{b}}\times 100\%$$

A_c_, A_s_ and A_b_ are the absorbances of the control well, the experimental well and the blank well, respectively.

#### 4.3.5. In Vivo Targeting Studies of Drug-Loaded Liposomes

Establishment of a mouse breast cancer model:4T-1 breast cancer cells were cultured to the logarithmic growth phase, collected and washed with PBS, and the cells were resuspended in medium to adjust the cell concentration to 1 × 10^7^ cells/mL. Under sterile conditions, 0.2 mL of cell suspension was injected under the skin of the forelimb axilla of the mouse using a sterile syringe to form a small circular bulge after injection. Monitor the formation and growth of the tumor after vaccination, do not touch the bulge with your hands three days before vaccination, and measure the tumor volume after the formation of solid tumors for about a week, and the volume formula is as follows:

V = (ab^2^)/2 (8)

a: the longest diameter of the tumor, b: the longest transverse diameter perpendicular to the longest diameter.

Preparation of Dir/Cu-Cur DTLPs of curcumin–copper chelate containing Dir: Liposomes were prepared by film dispersion, and after dissolving the membrane material and the organic reagent for medicine, 300 μL of Dir mother liquor was added, the organic reagent was waved in a round-bottom flask to obtain a film, 5 mL of normal saline was added after vacuum drying to remove the remaining organic reagents, and the film was dissolved in a magnetic stirrer at 400 rpm and 25 °C for 0.5 h, and the membrane was filtered after ultrasonic breaking for 2 min. After preparation, break the emulsion and use an enzyme-linked immunosorbent assay (ELISA) reader to determine the encapsulation efficiency of Dir in the liposomes.

Preparation of Dir/Cu-Cur LPs containing curcumin–copper chelates: Same as the preparation of Dir/Cu-Cur DTLPs, the membrane prescription was changed to EPC:Cholesterol:EPG:DSPE-mPEG2k = 120:20:20:60. After preparation, break the emulsion and use an enzyme-linked immunosorbent assay (ELISA) reader to determine the encapsulation efficiency of Dir in the liposomes.

Preparation of Dir sol group: Dilute the Dir mother liquor with normal saline to about 80 μg/mL, and use a microplate reader to determine the content.

When the tumor volume reached about 200 mm^3^ size, the mice were randomly divided into 3 groups of 3 in each group. The grouping was as follows: Dir solution group, Dir/Cu-Cur LPs group, and Dir/Cu-Cur DTLPs group. After the mouse were numbered, the dose was converted to 1 mg/kg of Dir per mouse, and in vivo imaging experiments were carried out in the form of tail vein injection. Each mouse was injected with 0.2 mL of Dir solution or liposomal formulation intravenously, and the administration time was recorded, and the mice were anesthetized with isoflurane at 1 h, 4 h, 12 h and 24 h after administration, respectively. Background scan and fluorescence scan were performed, respectively, and the detection conditions were set for excitation wavelength 710 nm and emission wavelength 800 nm. After each shooting, the mice were placed on a cryogenic heating pad and put back in the animal box after they woke up, and after the shooting tasks at all time points were completed, the mice were sacrificed, and their hearts, liver, spleen, lungs, kidneys and tumors were dissected, respectively, the surface was gently rinsed with normal saline, and the above organs and tumor tissues were placed on the black cardboard in a certain order, and then placed in the vivo imaging system for photographic measurement, which was used to analyze the distribution strength of Dir in various organs and tumor tissues of different groups.

#### 4.3.6. In Vivo Anti-Tumor Experimental Study Targeting Drug-Loaded Liposomes

Experimental grouping and dosing regimen: Using 4T-1 breast cancer cells for tumor modeling, each mouse successfully implanted with tumors was individually identified. When tumors reached approximately 100 mm^3^ in size, mice were randomly assigned to six groups of six mice each. Specific identification numbers for each group were recorded for subsequent experiments. The specific grouping details are as follows: paclitaxel solution group (PTX group), curcumin solution group (Cur sol), curcumin ordinary liposome group (Cur-LPs), curcumin–copper chelate ordinary liposome group (Cu-Cur LPs group), curcumin–copper chelate targeted liposome group (Cu-Cur DTLPs), saline group (Saline group),of which the first five groups were the preparation group and the last group was the control group. The mice in each preparation group were weighed and the dose was calculated according to body weight, and the volume was 0.2 mL, which was administered by tail vein injection, and the same 0.2 mL was injected in the saline group. The dosage of PTX group and Cur sol group was 5 mg/kg, and the dosage of Cur-LPs group, Cu-Cur LPs group and Cu-Cur DTLPs group was 5 mg/kg in curcumin terms, and the dosing cycle was 12 days, administered once every two days.

Tumor growth assessment: at the time of each administration, the tumor-bearing mice in each group were carefully observed. Pay close attention to their dietary intake, including the amount of food they eat, the frequency of eating, and record their daily activities, including their range of activities, activity, and whether there are any abnormal behaviors. At the same time, each mouse was weighed and the weight gain was calculated, which provided a reference for the toxicity of the preparation through the weight gain curve over time. For the tumor growth status of each mouse, the tumor volume is regularly measured and the data is recorded in detail for subsequent scientific analysis of tumor growth trends.

The weight gain formula is as follows:

(9) $$\mathrm{Weight}\;\mathrm{gain}\;(\%)\;=\;\frac{\mathrm{Current}\;\mathrm{Weight}-\mathrm{Initial}\;\mathrm{weight}}{\mathrm{Initial}\;\mathrm{weight}}\times 100\%$$

The first day of administration was set as Day 0, followed by the measurement of the longest diameter (a) and longest transverse diameter (b) of mouse tumors on days 0, 2, 4, 6, 8, 10, and 12, respectively, the volume of the tumor (V = (ab^2^)/2), and finally the curves of tumor volume and time change. On the second day after the last dose, the mice were removed from the animal culture center and transferred to the operation room, the blood of the mice was taken and sacrificed, the tumor tissue of each mouse was removed and weighed, and the relative tumor quality (%) and tumor growth inhibition rate (TGI) were calculated after recording the relevant data.

(10) $$\mathrm{Relative}\;\mathrm{Tumor}\;\mathrm{Mass}\;(\%)=\frac{\mathrm{Tumor}\;\mathrm{weight}\;\mathrm{after}\;12\;\mathrm{days}}{\mathrm{Average}\;\mathrm{tumor}\;\mathrm{weight}\;\mathrm{in}\;\mathrm{the}\;\mathrm{control}\;\mathrm{group}\;\mathrm{after}\;12\;\mathrm{days}\;\mathrm{of}\;\mathrm{administration}}\times 100\%$$

(11) $$\mathrm{TGI}=(1-\frac{T-T_{0}}{C-C_{0}})\times 100\%$$

T and T_0_ were the average tumor volume in the treatment group on the last day of administration and before administration, respectively. C and C_0_ were the average tumor volume of the control group on the last day of administration and before administration, respectively.

##### Safety Evaluation

1. Organ index of mice

At the end of the administration cycle, the tumor-bearing mice are sacrificed, and then the main organs such as the heart, liver, spleen, lungs, and kidneys are dissected and weighed, and the ratio of the weight of each organ of the mouse to the weight of the mouse is the organ index, which is shown below.

(12) $$\mathrm{Organ}\;\mathrm{Index}=\frac{\mathrm{Organ}\;\mathrm{weight}\;(\mathrm{mg})}{\mathrm{Mouse}\;\mathrm{body}\;\mathrm{weight}\;(g)}$$

2. Liver and kidney function tests

By determining specific biochemical markers in serum, the functional status of different organs and systems can be systematically assessed to determine the degree of toxicity. ALT (alanine aminotransferase) and AST (aspartate aminotransferase) indicators are used in liver toxicity assessments, and CRE (creatinine) indicators are used in renal toxicity assessments.

3. Routine blood examination of mice

Tumor models or therapeutic agents may inhibit bone marrow hematopoietic function, resulting in cytopenia, white blood cell count (WBC) to assess immune system status, red blood cell count (RBC) and hemoglobin (HGB) to assess anemia, and platelet count (PLT) to assess coagulation function.

4. H&E staining of major organs

H&E staining was performed on the main organs of mice (such as heart, liver, spleen, lung, and kidney) to compare the differences in tissue staining between the administration group and the control group.

##### Research on the Mechanism of Antitumor Action of the Preparation

1. Copper ion accumulation analysis

The accumulation of copper ion concentration is the key to the occurrence of copper death, and the copper ion levels in serum, liver and tumor tissue in different mouse groups were detected, and the differences in copper accumulation between different groups were compared.

2. Immunohistochemistry (IHC) staining analysis

Tumor tissue samples were fixed with 4% paraformaldehyde for 24 h, paraffin-embedded, and 4 μm thick sections were prepared. After dewaxing and hydration, the sections were decorated with sodium citrate buffer (pH 6.0, 95 °C high-pressure repair for 15 min), followed by 3% H_2_O_2_ to block endogenous peroxidase activity, and 5% goat serum for 30 min at room temperature to reduce non-specific binding. Rabbit-derived anti-DLAT monoclonal antibody (1:500) and rabbit-derived anti-FDX1 monoclonal antibody (1:300) were added to drop, incubated overnight at 4 °C, and incubated at room temperature with HRP-labeled goat anti-rabbit secondary antibody (1:500) for 1 h the next day. Each group of sections was randomly selected at 10× magnification, and the percentage of DLAT or FDX1-positive areas in the field was calculated by ImageJ 1.54g software threshold segmentation method, and the data were expressed as Mean ± SD, and the differences between groups were analyzed by univariate ANOVA and Tukey multiple comparison test, and the significance threshold was set as * *p* < 0.05, ** *p* < 0.01, ** *p* < 0.001.

3. Changes in metabolite levels

The TCA cycle is an important metabolic pathway in the mitochondria, responsible for the production of energy and metabolic intermediates, citric acid, succinic acid and α-ketoglutarate are intermediate products of the TCA cycle, the changes in the levels of these metabolites are closely related to copper death, detect the relevant content in serum and tumor tissue, respectively, and analyze how copper affects various steps of the TCA cycle.

4. Tumor tissue H&E staining and TUNEL staining

H&E staining and TUNEL staining of mouse tumor tissues can clearly show the morphological structure of tissue cells, including the size, shape, and arrangement of cells, the morphology, size, and chromatin distribution of nuclei, so as to observe and compare the morphological characteristics of normal tissues and tumor tissues, and provide a basis for subsequent pathological analysis.

Statistical analysis: Data analysis and plotting were performed using Graphpad Prism 10.1.2, and all results were expressed as mean (Mean) ± standard deviation (SD). Statistical significance was analyzed using significance *t*-test and one-way analysis of variance (ANOVA) (* represents *p* < 0.05, ** represents *p* < 0.01, *** represents *p* < 0.001).

## 5. Conclusions

Based on the copper death mechanism and the synergistic effects between curcumin and copper, this study developed a curcumin–copper chelate (Cu-Cur) and achieved copper accumulation at tumor sites through a targeted liposomal delivery system. A series of experiments demonstrated that Cu-Cur DTLPs significantly outperformed other curcumin formulations in reducing tumor volume and mass while enhancing tumor suppression rates. Concurrently, Cu-Cur DTLPs exhibited minimal toxicity to major organs and demonstrated favorable safety profiles. Their mechanism of action primarily involves inducing copper accumulation in tumor tissues, disrupting the TCA cycle within tumor cells, and ultimately causing metabolic dysfunction and cell death.

## Figures and Tables

**Table 1:** Summary of EPR Parameters.

Sample	Main Peak (G)	g//	Second Peak (G)	g⊥	*A*// (mT)	*A*⊥ (mT)
Filtrate	3234	2.10	3056	2.22	51	54
Filter residue	3220	2.03	3048	2.15	53	54

**Table 2:** Melting Point Measurement Results.

Measurement Count	Sample Melting Point (°C)	
	Filtrate Product	Filter Residue
1	137–145	260
2	137–145	259
3	136–143	258

**Table 3:** Particle Size, PDI, and Potential of Cu-Cur DTLPs, Cu-Cur LPs, and Cur-LPs. Data are shown as mean ± SD (*n* = 3).

Liposome	Size (nm)	PDI	Zeta (mV)
Cu-Cur DTLPs	104.4 ± 2.84	0.214 ± 0.007	−19.1 ± 0.76
Cu-Cur LPs	117.5 ± 1.06	0.261 ± 0.010	−17.4 ± 1.79
Cur-LPs	126.3 ± 0.91	0.25 ± 0.002	−17.8 ± 0.91

**Table 4:** Encapsulation Efficiency and Drug Loading Capacity. Data are shown as mean ± SD (*n* = 3).

Sample	EE (%)	Cu-Cur DLE (%)
Cu-Cur DTLPs	96.41 ± 0.75	1.21 ± 0.02
Cu-Cur LPs	83.62 ± 1.54	0.92 ± 0.02
Cur-LPs	52.38 ± 1.30	0.85 ± 0.03

**Table 5:** Iodide Content in DIR Preparations by Formulation Group.

Group	Concentration (μg/mL)	Average Preparation Concentration (μg/mL)	Average Drug Concentration (μg/mL)	Average Encapsulation Rate (%)
Dir	78.53			
	78.97	78.68	78.68	-
	78.53			
Dir/Cu-Cur LPs	71.56			
	70.64	73.91	73.91	83.74
	79.52			
Dir/Cu- Cur DTLPs	74.92			
	87.39	81.18	73.18	87.90
	81.23			

## Data Availability

The original contributions presented in this study are included in the article. Further inquiries can be directed to the corresponding author.

## Abbreviations

The following abbreviations are used in this manuscript:

ALT	Alanine Aminotransferase
AST	Aspartate Aminotransferase
ATP	Adenosine Triphosphate
CA	Citric Acid
CRE	Creatinine
Cur	Curcumin
DIR	1,1′-dioctadecyl-3,3,3′,3′-tetramethylindodicarbocyanine iodide
DLAT	Dihydrolipoyl transacetylase
EDTA	Ethylenediaminetetraacetic acid
ER	Estrogen Receptor
FBS	Fetal Bovine Serum
H&E	Hematoxylin and Eosin
HER2	Human Epidermal Growth Factor
HGB	Hemoglobin
logP	Partition coefficient
LPs	Liposomes
MTT	3-(4,5-dimethylthiazol-2-yl)-2,5-diphenyltetrazolium bromide
PTX	Paclitaxel
RPMI-1640	Roswell Park Memorial Institute 1640 medium
TGI	Tumor Growth Inhibition
TNBC	Triple-Negative Breast Cancer
TUNEL	Terminal deoxynucleotidyl transferase dUTP nick end labeling
WBC	White Blood Cell
α-KG	α-Ketoglutaric acid

## Appendix A

**Table A1:** logP Values for Cur.

No. /n-o	A.	Cn-o	Orig. Cn-o	No. /w	A.	Cw	Pow = Cn-o/Cw	logP	Avg.logP
1-1	1.2764	10.17	111.92	1-1	0.0053	0.06	1803.58	3.26	
1-2	1.275	10.16	111.79	1-2	0.0024	0.04	2867.86	3.46	
1-3	1.2759	10.17	111.87	1-3	0.0014	0.03	3605.74	3.56	
2-1	1.0554	8.42	92.58	2-1	0.0025	0.04	2327.38	3.37	
2-2	1.0566	8.43	92.68	2-2	0.0009	0.03	3426.50	3.53	3.38
2-3	1.0613	8.46	93.09	2-3	0.0008	0.03	3546.00	3.55	
3-1	1.1126	8.87	97.58	3-1	0.0068	0.07	1318.94	3.12	
3-2	1.132	9.03	99.28	3-2	0.0047	0.06	1733.26	3.24	
3-3	1.1058	8.82	96.99	3-3	0.0036	0.05	1998.57	3.30	

**Table A2:** logP Values for Cu-Cur.

No. /n-o	A.	Cn-o	Orig. Cn-o	No. /w	A.	Cw	Pow = Cn-o/Cw	logP	Avg.logP
1-1	0.5106	7.53	82.88	1-1	0.0841	1.23	67.12	1.83	
1-2	0.5167	7.62	83.87	1-2	0.092	1.35	62.06	1.79	
1-3	0.5241	7.73	85.08	1-3	0.0945	1.39	61.27	1.79	
2-1	0.6999	10.33	113.64	2-1	0.049	0.72	158.63	2.20	
2-2	0.6999	10.33	113.64	2-2	0.0497	0.73	156.37	2.19	2.09
2-3	0.7008	10.34	113.79	2-3	0.0514	0.75	151.34	2.18	
3-1	0.7792	11.50	126.52	3-1	0.0441	0.64	196.46	2.29	
3-2	0.7759	11.45	125.99	3-2	0.0463	0.68	186.23	2.27	
3-3	0.7744	11.43	125.74	3-3	0.0468	0.68	183.86	2.26	

**Table A3:** Particle Size Changes in Cu-Cur DTLPs Over 28 Days (*n* = 3).

Time (Day)	Size (nm)		
	Liposome 1	Liposome 2	Liposome 3
1	106.7	108.8	100.4
2	108.8	109.4	101.7
3	116	116.2	104.9
5	112.6	112.6	108.6
7	111.8	111.5	108.7
9	111.7	110.9	109.3
11	113.4	110.8	108.1
13	111.1	110.6	108.3
21	110.7	108.4	105
28	109.8	104.1	104.6
Mean ± SD	111.26 ± 2.55	110.33 ± 3.11	105.96 ± 3.13
RSD (%)	2.29	2.82	2.96

**Table A4:** Potential Changes in Cu-Cur DTLPs Over 28 Days (*n* = 3).

Time (Day)	Zeta (mV)		
	Liposome 1	Liposome 2	Liposome 3
1	−18.4	−19.0	−19.3
2	−18.9	−19.5	−18.0
3	−17.6	−18.4	−18.8
5	−17.7	−18.3	−18.1
7	−18.1	−17.9	−18.8
9	−18.3	−18.6	−18.6
11	−17.5	−18.4	−18.3
13	−18.5	−17.8	−19.3
21	−18.6	−18.1	−18.0
28	−18.6	−18.0	−18.0
Mean ± SD	−18.2 ± 0.48	−18.4 ± 0.53	−18.52 ± 0.52
RSD (%)	2.62	2.85	2.79
